# Multi-study reanalysis of 2,213 acute myeloid leukemia patients reveals age- and sex-dependent gene expression signatures

**DOI:** 10.1038/s41598-019-48872-0

**Published:** 2019-08-27

**Authors:** Raeuf Roushangar, George I. Mias

**Affiliations:** 1 Department of Biochemistry and Molecular Biology, Michigan State University, East Lansing, MI 48824 USA; 2 Institute for Quantitative Health Science and Engineering, Michigan State University, East Lansing, MI 48824 USA

**Keywords:** Acute myeloid leukaemia, Gene expression, Transcriptomics

## Abstract

In 2019 it is estimated that more than 21,000 new acute myeloid leukemia (AML) patients will be diagnosed in the United States, and nearly 11,000 are expected to die from the disease. AML is primarily diagnosed among the elderly (median 68 years old at diagnosis). Prognoses have significantly improved for younger patients, but as much as 70% of patients over 60 years old will die within a year of diagnosis. In this study, we conducted a reanalysis of 2,213 acute myeloid leukemia patients compared to 548 healthy individuals, using curated publicly available microarray gene expression data. We carried out an analysis of normalized batch corrected data, using a linear model that included considerations for disease, age, sex, and tissue. We identified 974 differentially expressed probe sets and 4 significant pathways associated with AML. Additionally, we identified 375 age- and 70 sex-related probe set expression signatures relevant to AML. Finally, we trained a k nearest neighbors model to classify AML and healthy subjects with 90.9% accuracy. Our findings provide a new reanalysis of public datasets, that enabled the identification of new gene sets relevant to AML that can potentially be used in future experiments and possible stratified disease diagnostics.

## Introduction

Acute myeloid leukemia (AML) is a heterogeneous malignant disease of the hematopoietic system myeloid cell lineage^1–5^. AML is best characterized by terminal differentiation in normal blood cells and excessive production and release of cells at various stages of incomplete maturation (leukemia cells). As a result of this faster than normal, and uncontrolled growth of leukemia cells, healthy myeloid precursors involved in hematopoiesis are suppressed, and ultimately can soar to death within months from diagnosis if untreated^1,6^. AML accounts for 70% of myeloid leukemia and nearly 80% of acute leukemia cases, making it the most common form of both myeloid and acute leukemia^1,7^. The number of new AML cases is increasing each year – in 2019 alone, an estimated 21,450 new AML patients will be diagnosed, and nearly 10,920 are expected to die from the disease^8^.

According to the 2016 World Health Organization (WHO) newly revised myeloid neoplasms and acute leukemia classification system^9^, AML prognosis criteria for classification are highly dependent on the presence of chromosomal abnormalities, including chromosomal deletions, duplications, translocations, inversions, and gene fusions. AML is diagnosed predominantly through microscopic, cytogenetic, and molecular genetic analyses of patients’ blood, and/or bone marrow samples. Microscopic examination may be used to detect distinctive features (e.g. Auer rods) in cell morphology, cytogenetic analysis to identify chromosomal structural aberrations (e.g., t(8;21), inv(16), t(16;16), or t(9;11)), and molecular genetic analysis to identify gene fusion (e.g., RUNX1-RUNX1T1 and CBFB-MYH11), and mutations in genes frequently mutated in AML (e.g., NPM1, CEBPA, RUNX1, FLT3)^1,3,5,10–12^. Such cytogenetic and molecular genetic analyses are used to identify prognosis markers for classifying AML patients into three risk categories: favorable, intermediate, and unfavorable, currently based primarily on the European LeukemiaNet (ELN) 2017 classification^3,10^ (see Estey^3^ for a recent review, including ELN assessments). A large group of AML patients present normal karyotypes and lack chromosomal abnormalities^3,5,10,11,13^. These patients are classified as intermediate risk, and often have heterogeneous clinical outcome with standard therapy with risk of AML relapse^3,5,14^.

Additionally, AML prognosis worsens with age, and older patients respond less to current treatments, with poorer clinical outcomes compared to younger patients^15,16^. AML can occur in people of all ages but is primarily diagnosed among the elderly (>60 years old), with a median age of 68 years at diagnosis^8^. Recent advances in AML biology have expanded our understanding of its complex genetic landscape, and led to significant improvement in prognoses and therapeutic strategy for younger patients^2,16^. For elderly patients, prognoses remain grim and the main therapeutic strategy, remission induction therapy followed by an intensive consolidation phase (post-remission), had remained nearly unchanged over the past three decades^1,2,4,5,10,16,17^. More recently, however, new therapeutic agents have been approved for older AML patients^4,5^, and these include venetoclax (combined with decitabine or azacitidine)^18^, midostaurin (combined with standard chemotherapy)^19^, and gilteritinib^20^. It is expected that the new therapeutic agents will improve prognosis for older AML patients (where in the past up to 70% of AML patients aged 65 or older were reported to die within a year following diagnosis^21^). While it is apparent that the nature of AML changes with age, still little is known about the extent of these associations and how they vary with patient age^2,22,23^, and current indications from ELN and the National Comprehensive Cancer Network (NCCN) essentially consider age as a surrogate variable that is used only in conjunction with other treatment‐related mortality factors^3,5,10^. Taking into consideration age in the identification of changes in AML global gene expression may lead to improved early diagnosis and improvement in treatment approaches for elderly patients. To further complicate matters, AML has multiple driver mutations and competing clones that evolve over time, making it a very dynamic disease^13,24^.

Multiple gene expression analyses of AML have been carried out, 25 of which have been systematically compared by Miller and Stamatoyannopoulos^25^, who analyzed information on 4,918 genes, and identified 25 genes reported across multiple studies, with potential prognostic features. In this study, we performed a comprehensive gene expression analysis of 2,213 AML patients and 548 healthy subjects, by re-analyzing publicly available gene expression microarray data from 37 curated studies (a reanalysis following strict inclusion criteria) and identified disease-, age- and sex-related gene expression changes associated with AML. The differentially expressed gene sets were associated to signaling pathways relevant in AML, and also used to train and test a predictive model of AML or healthy status. We believe that our results may lead to improved AML early detection, and diagnostic testing with target genes, which collectively can potentially serve as age- and sex-dependent biomarkers for AML prognosis, as well as new treatment targets with mechanisms of action different from those used in conventional chemotherapy.

## Results

### Data curation and gene expression pre-processing

**Figure 1:**
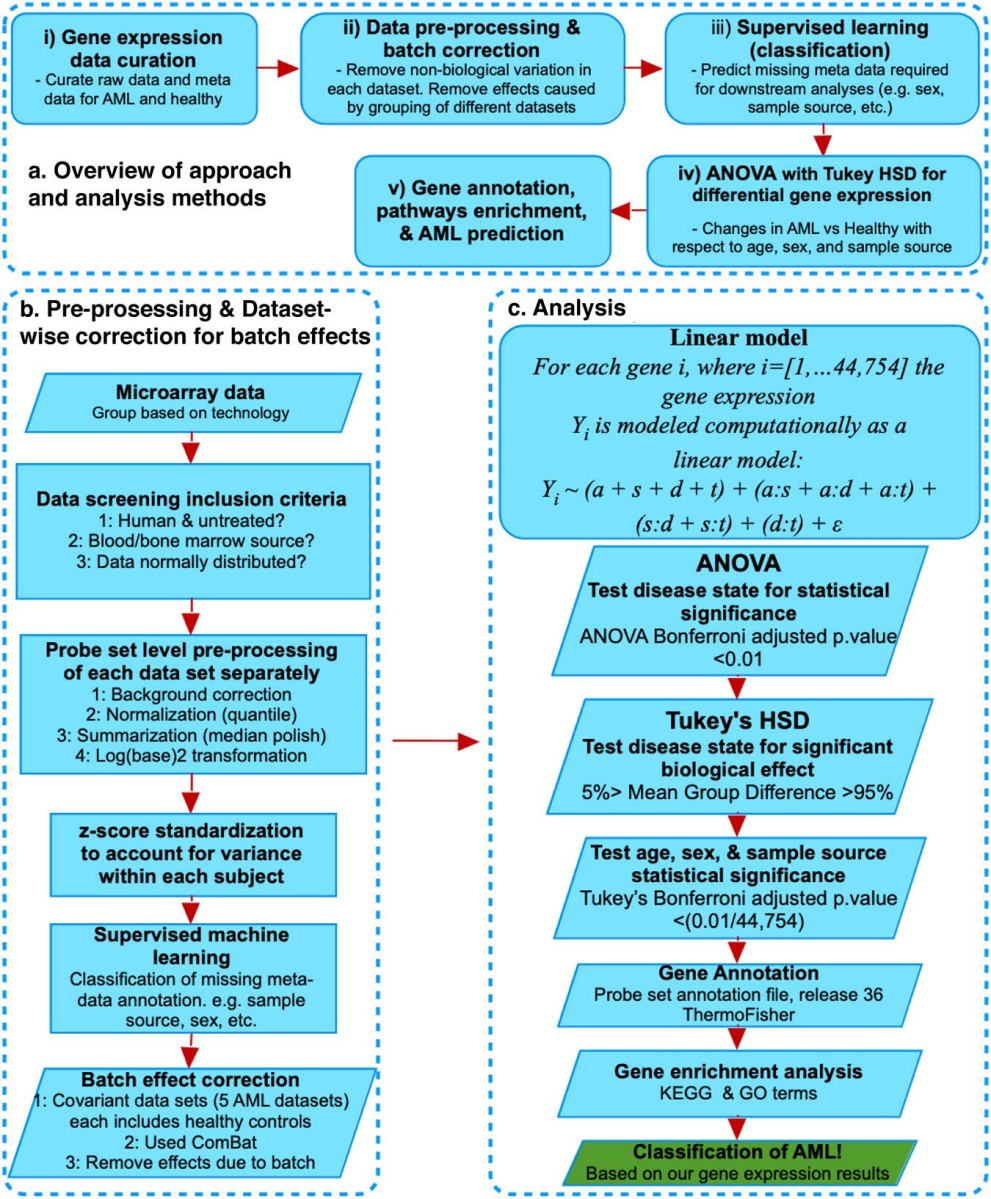
General approach, data curation, and analysis workflow summary. The flowchart shows in (**a**) the five main steps that summarize our method of approach for our study, and in (**b**) the curation and screening criteria for raw gene expression and annotation data files curation, data pre-processing, supervised machine learning for missing metadata prediction, and batch effects correction. (**c**) The analysis included a linear model analysis of variance (ANOVA) coupled with Tukey’s Honestly Significant Difference (HSD) post-hoc tests, and KEGG pathway and GO enrichment. Finally, we performed a machine learning classification of AML based on our findings.

We searched the Gene Expression Omnibus (GEO) public repository, based on our systematic workflow and inclusion criteria, Fig. 1a,b. Overall, 2,132 datasets were screened, and 643 selected (577 were excluded as non-Affymetrix, various platform arrays). From the 66 remaining corresponding studies, 34 were excluded due to: lack of metadata, using non-peripheral blood or non-bone marrow tissues, or being cell line or cell-type specific, or analyzing treated subjects. After this curation we obtained 34 age-annotated gene expression datasets from 32 different studies covering 2,213 AML patients and 548 healthy individuals. These 34 datasets were reanalyzed, starting from raw microarray data, to perform a gene expression analysis of variance and functional pathway enrichment analysis (see online Methods). Table 1 provides a description of each dataset with a sub-table summary of all curated data used in this study. After pre-processing each individual dataset separately, Fig. 1b, we performed the statistical analysis on 44,754 probe sets which were common across all samples (Affymetrix expression microarray data).

**Table 1:** Summary table gene expression datasets used in this study.

Author, Year		GEO accession		Disease Status*		Affymetrix platform id: Number of samples used & Sample source*	Refs*
**(A) Curated datasets used in linear model analysis (34 datasets from 32 studies)**							
Zatkova *et al*., 2009		GSE10258		AML		GPL570: 8 BM	^68^
Tomasson *et al*., 2008		GSE10358		AML		GPL570: 300 BM	^69^
Metzeler *et al*., 2008		GSE12417		AML		GPL570: 73 BM & 5 PB GPL96/97: 160 BM & 2PB	^55^
Wouters *et al*., 2009, Taskesen *et al*., 2011		GSE14468		AML		GPL570: 482 BM & 43 PB	^70, 71^
Figueroa *et al*., 2009		GSE14479		AML		GPL570: 16 BM	^72^
Klein *et al*., 2009		GSE15434		AML		GPL570: 231 BM & 20 PB	^73^
Lück *et al*., 2011		GSE29883		AML		GPL570: 10 BM & 2 PB	^74^
Li *et al*., 2013, Herold *et al*., 2014, Janke *et al*., 2014, Jiang *et al*., 2016		GSE37642		AML		GPL570: 140 BM GPL96/97: 422 BM	^56– 59^
Bullinger *et al*., 2014		GSE39363		AML		GPL570: 11 BM & 2 PB	NYP
Opel *et al*., 2015		GSE46819		AML		GPL570: 8 BM & 4 PB	^75^
TCGA *et al*., 2015		GSE68833		AML		GPL570: 183 BM	NYP
Cao *et al*., 2016		GSE69565		AML		GPL570: 12 PB	^76^
Bohl *et al*., 2016		GSE84334		AML		GPL570: 25 BM & 20 PB	NYP
Li *et al*., 2011		GSE23025		AML		GPL570: 21 BM & 13 PB	^77^
Warren *et al*., 2009		GSE11375		Healthy		GPL570: 26 PB	^78^
Green *et al*., 2009		GSE14845		Healthy		GPL570: 1 PB	NYP
Wu *et al*., 2012		GSE15932		Healthy		GPL570: 8 PB	NYP
Karlovich *et al*., 2009		GSE16028		Healthy		GPL570: 22 PB	^79^
Krug *et al*., 2011		GSE17114		Healthy		GPL570: 14 PB	NYP
Kong *et al*., 2012		GSE18123		Healthy		GPL570: 17 PB	^80^
Sharma *et al*., 2009		GSE18781		Healthy		GPL570: 25 PB	^81^
Rosell *et al*., 2011		GSE25414		Healthy		GPL570: 12 PB	^82^
Schmidt *et al*., 2006		GSE2842		Healthy		GPL570: 2 PB	^83^
Meng *et al*., 2015		GSE71226		Healthy		GPL570: 3 PB	NYP
Tasaki *et al*., 2017		GSE84844		Healthy		GPL570: 30 PB	^84^
Leday *et al*., 2018		GSE98793		Healthy		GPL570: 64 PB	^85^
Shamir *et al*., 2017		GSE99039		Healthy		GPL570: 121 PB	^86^
Tasaki *et al*., 2018		GSE93272		Healthy		GPL570: 35 PB	^87^
Clelland *et al*., 2013		GSE46449		Healthy		GPL570: 24 PB	^88^
Lauwerys *et al*., 2013 Ducreux *et al*., 2016		GSE39088		Healthy		GPL570: 46 PB	^89, 90^
Xiao *et al*., 2011		GSE36809		Healthy		GPL570: 35 PB	^91^
Zhou *et al*., 2010		GSE19743		Healthy		GPL570: 63 PB	^92^
**(B) Covariate datasets (used for batch correction and for testing predictive models)**							
*Jiang et al*., 2018^#^		GSE107968^*^		2 AML; 1 Healthy		GPL570: 3 BM	NYP
*Greiner et al*., 2015^#^		GSE68172^*^		20 AML; 5 Healthy		GPL570: 25 PB	^64^
*Majeti et al*., 2009^#^		GSE17054^*^		9 AML; 4 Healthy		GPL570: 13 BM	^65^
*Bacher et al*., 2012^#^		GSE33223^*^		20 AML; 10 Healthy		GPL570: 30 PB	^66^
*Mills et al*., 2009^#^		GSE15061^*^		404 AML; 138 Healthy		GPL570: 542 BM	^67^
**(C) Analysis datasets summary statistics**							
**Disease state**		**Sample source**		**Affymetrix platform id**		**Unique probe sets**	
AML	Healthy	BM	PB	GPL570	GPL96/97	GPL570	GPL96/97
2,213	548	2,090	671	2,177	584	54,675	44,760

Summary of datasets used in our analysis and disease classification. *GEO, Gene Expression Omnibus; AML, acute myeloid leukemia; Refs., references; NYP, not yet published; GPL570, Affymetrix Human Genome U133 Plus 2.0 Array; GPL96, Affymetrix Human Genome U133A Array; GPL97, Affymetrix Human Genome U133B Array; BM, Bone Marrow; PB, Peripheral Blood.

### Classification of missing metadata annotation

Following the data curation step, 805 arrays (802 AML and 3 healthy) of 2,761 curated data were found to be missing sex annotation, and 737 arrays (all AML patients) were missing sample source annotation (i.e. whether the tissue from which RNA had been extracted was either bone marrow [BM] or peripheral blood [PB]). To predict the missing sex and sample source annotations, we trained and validated a logistic regression (LR) classification model. The prediction of missing annotations for these arrays was essential in our study, to increase the sample size, and statistical power^26^. The trained models were cross-validated using our annotated preprocessed expression data, and were 96 ± 8% and 96.7 ± 4% accurate for sex and sample source predictions respectively (see Supplementary Table S1 and Fig. S1 for additional LR model performance metrics). Model training, parameters used in training, and validation for this analysis are discussed in the Online Methods. The results from classification for missing annotation were used for the downstream analysis of gene expression variability, and are presented in Supplementary Files S1 and S2 for sample source and sex annotations respectively.

### Batch correction

The different datasets we curated for this study did not include within-study healthy controls, which would limit analysis of variance, and particularly the ability to separate biological from batch effects. To address this, we implemented an iterative batch effect correction approach, essentially employing a weight-based method for correcting batch effects – here we use the term “dataset-wise” batch effect correction for this approach. Assuming the batch effects due to each dataset are a function of the number of samples in the dataset (weight), normalizing sets of unevenly sized datasets may lead to an unbalanced batch correction. We used 5 additional datasets as a reference set, which we refer to as “covariate” hereafter. Each of the covariate reference datasets included within-study healthy controls. All 5 datasets together consisted of a total 613 arrays (455 AML and 158 healthy) (Table 1), and were pre-processed exactly as our curated datasets. Each of the remaining datasets was batch corrected with respect to the combined covariate datasets reference using ComBat^27^. After this dataset-wise correction, the 5 covariate reference datasets were removed, and our expression data were clustered using principal component analysis (PCA), to visually examine the effect of covariate reference datasets on distributing the batch weight during batch correction (Supplementary Fig. S2).

### Analysis 1: Gene expression analysis and enrichment analysis of AML disease state compared to healthy individuals

#### Gene expression analysis of AML disease state

**Figure 2:**
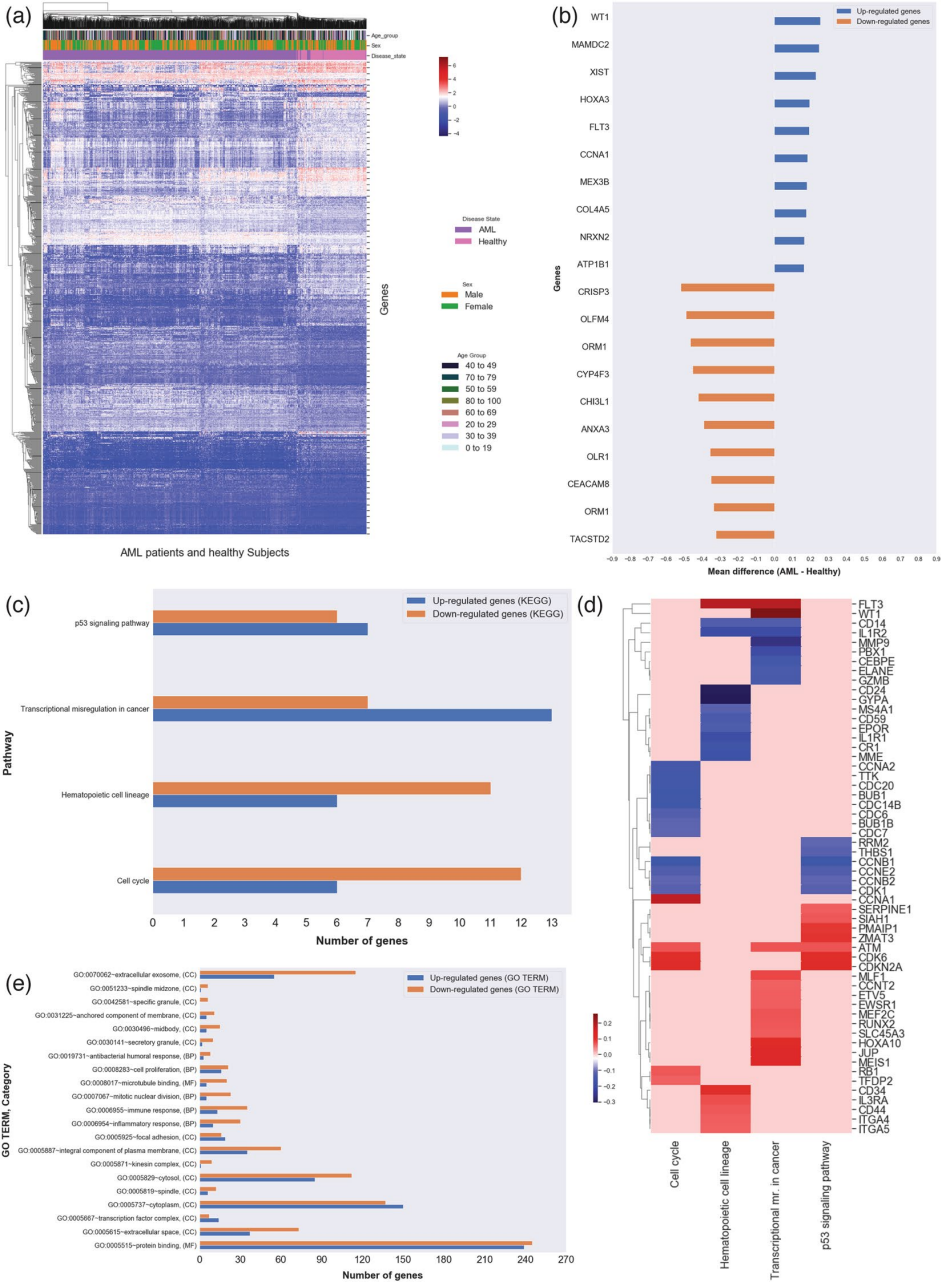
Functional classification of DEPS from AML analysis and associated KEGG and GO enrichment analysis. For all panels, normalized values are represented in blue for down-regulation and red for up-regulation, while light red/gray represents no reported specific direction. (**a**) Heatmap of 974 DEPS (rows) on 2,761 arrays (columns) including 2213 AML patients and 548 healthy individuals from AML analysis, using unsupervised hierarchical clustering and Euclidean distance for clustering. The age of each individual is illustrated in the color bar on the top (dark green for old and light blue for young). The disease state (AML vs healthy), sex of each subject and age-groups are also represented in color bars on the top. (**b**) Horizontal bar plot of the top 10 DEPS (gene symbols on vertical axis) from AML analysis with mean difference values between AML and healthy (horizontal axis). Enrichment analysis identified 4 KEGG signaling pathways (**c**) for our AML DEPS, also visualized as a heatmap (**d**) of DEPS mean difference values between AML and healthy DEPS (rows) identified in these 4 KEGG signaling pathways (columns). The GO enrichment analysis results are summarized in (**e**).

Following batch correction, we performed an analysis of differential expression (DE) on the 34 curated datasets including 2,213 AML patients and 548 healthy controls. Analysis of Variance (ANOVA)^28–30^ was performed according to a linear model (see Online Methods), including factors for age, sex, and sample source (to account for tissue differences between AML and healthy), as well as binary interactions thereof. To avoid assumptions on averaging over multiple probe sets corresponding to the same gene symbol, we analyzed probe sets with the linear model. We identified 974 statistically significant differentially expressed probe sets (DEPS) (corresponding to 964 unique gene symbols) for AML, based on post-hoc analyses (Tukey’s Honestly Significant Difference (HSD) tests implemented in R, with adjusted p-value < 0.01), in conjunction with a two-tailed 5% quantile selection^31^ based on the mean difference distribution between AML-healthy group comparisons across probe sets (to identify largest biological effects). The heatmap (Fig. 2a) shows the hierarchical clustering of gene expression from the 974 DEPS, including 487 up- and 487 down-regulated with respect to AML as compared to healthy. From this analysis, WT1 (Wilms tumor 1) with mean difference of 0.26 and adjusted p-value < 4.11 × 10^−11^ was the most DE up-regulated gene while CRISP3 (cysteine-rich secretory protein 3) with mean difference of −0.52 and adjusted p-value < 4.11 × 10^−11^ was the least DE gene. Figure 2b shows the top 10 up- and down-regulated DEPS with corresponding gene symbols, that resulted from this analysis (also listed in Table 2, including mean difference and p-adjusted values from post-hoc analysis using Tukey’s HSD tests). The entire list of all 974 DEPS can be found as Supplementary Table S2.

**Table 2:** Top 10 up- and down-regulated of DEPS in AML from disease state.

Up-regulated*			
DEG name	DEPS Gene Symbol	Tukey’s HSD Mean difference	p-adjusted value (HSD test in R)
Wilms tumor 1	WT1	0.255353	<4.11E-11
MAM domain containing 2	MAMDC2	0.248983	5.47E-09
X inactive specific transcript (non-protein coding)	XIST	0.230331	<4.11E-11
homeobox A3	HOXA3	0.195790	1.1E-06
fms-related tyrosine kinase 3	FLT3	0.193420	<4.11E-11
cyclin A1	CCNA1	0.185050	1.35E-07
mex-3 RNA binding family member B	MEX3B	0.181068	<4.11E-11
collagen, type IV, alpha 5	COL4A5	0.177721	1.7E-05
neurexin 2	NRXN2	0.166598	<4.11E-11
ATPase, Na+/K+ transporting, beta 1 polypeptide	ATP1B1	0.165197	5.47E-09
**Down-regulated**			
cysteine-rich secretory protein 3	CRISP3	−0.51965625	<4.11E-11
olfactomedin 4	OLFM4	−0.489845396	<4.11E-11
orosomucoid 1	ORM1	−0.465232864	<4.11E-11
cytochrome P450, family 4, subfamily F, polypeptide 3	CYP4F3	−0.453467442	<4.11E-11
chitinase 3-like 1 (cartilage glycoprotein-39)	CHI3L1	−0.421520435	<4.11E-11
annexin A3	ANXA3	−0.390688999	<4.11E-11
oxidized low density lipoprotein (lectin-like) receptor 1	OLR1	−0.35525472	<4.11E-11
carcinoembryonic antigen-related cell adhesion molecule 8	CEACAM8	−0.351181264	<4.11E-11
orosomucoid 1	ORM1	−0.336303304	<4.11E-11
tumor-associated calcium signal transducer 2	TACSTD2	−0.323939961	<4.11E-11

From the Post-hoc Tukey’s test, gene expression means difference value < 5% or >95% between AML and healthy (AML - healthy) were selected for biological effect from the statistically significant differentially expressed genes for disease state - based on the analysis of variance of all 2,761 cases (2,213 AML patients and 548 healthy controls). *Significant DEPS (gene symbols) are listed in descending order of the mean difference value comparisons for disease state.

#### (ii) Pathway and gene ontology enrichment analysis of DEPS

We carried out overrepresentation analysis in Kyoto Encyclopedia of Genes and Genomes (KEGG)^32–34^ signaling pathways, and Gene Ontology (GO) terms^35,36^ on all 974 DEPS, using the Database for Annotation, Visualization and Integrated Discovery (DAVID)^37,38^. Four KEGG signaling pathways were identified as enriched (Benjamini and Hochberg^39^ adjusted p-value < 0.05), including Hematopoietic cell lineage, Cell cycle, p53 signaling pathway, and Transcriptional misregulation in cancer. The 4 KEGG signaling pathways are summarized in Table 3 (see also Supplementary Fig. S3a–d), including unadjusted p-values, and Benjamini-Hochberg^39^ adjusted p-values. These signaling pathways were associated with 56 DEPS, including 27 up- and 29 down-regulated DEPS (Fig. 2c) - the heatmap of their mean differences (AML-healthy values) is shown in Fig. 2d. From our gene enrichment analysis for overrepresentation in GO terms, 21 GO terms were statistically significant (Benjamini and Hochberg^39^ adjusted p-value < 0.05), with 727 DE unique identities (335 up- and 392 down-regulated). GO terms included protein and microtubule binding for the molecular function (MF) category, inflammatory and immune responses, mitotic nuclear division, and cell proliferation response for the biological process (BP) category, and finally, cytoplasm, extracellular exosome, cytosol, extracellular space, integral component of plasma membrane immune response, and others, for the cellular component (CC) category (Fig. 2e). The complete list of the enrichment analysis results is shown in Supplementary Table S3.

**Table 3:** KEGG pathway analysis of DEPS from meta-analysis of 34 gene expression datasets.

AML Vs Healthy DEPS and associated signaling pathways					
Pathway	No. of genes*	Down-regulated	Up-regulated	p-value	p-value Benjamini adjusted
Hematopoietic cell lineage	11, 6	IL1R2, CD59, GYPA, MS4A1, EPOR, CD24, CD14, EPOR, IL1R1, MME, CR1	ITGA4, FLT3, CD34, IL3RA, ITGA5, CD44	2.3E-5	5.8E-3
Cell cycle	12, 6	CDC7, CDC6, CCNB1, CDC20, CCNA2, CCNE2, TTK, CDC14B’, CDK1, BUB1, CCNB2, BUB1B	RB1, CCNA1, CDK6, ATM, TFDP2, CDKN2A	1.4E-4	1.2E-2
p53 signaling pathway	6, 7	THBS1, CCNB1, CCNE2, CDK1, RRM2, CCNB2	SIAH1, CDK6, ATM, SERPINE1, CDKN2A, PMAIP1, ZMAT3	1.0E-4	1.3E-2
Transcriptional misregulation in cancer	7, 13	IL1R2, GZMB, CD14, ELANE, MMP9, CEBPE, PBX1	WT1, RUNX2, ETV5, MEIS1, JUP, EWSR1, ATM, HOXA10, MLF1, FLT3, CCNT2, MEF2C, SLC45A3	6.5E-4	4.1E-2
**AML sex relevant (male - female) DEPS & associated signaling pathways**					
Pathway	No. of genes*	**High in Females**		**High in Males**	
Hematopoietic cell lineage	1, 2	—		FLT3, CD34	
p53 signaling pathway	−, 1	—		PMAIP1	
Transcriptional misregulation in cancer	−, 1	MS4A1		FLT3	

Enrichment analysis was done using 974 DEPS, including KEGG enrichment analysis identified 4 statistically significant pathways from AML Vs Healthy analysis, shown with overlaps with sex-specific analysis.

*Up and down regulated genes displayed.

### Analysis 2. Gene expression analysis and enrichment analysis of sex- and age-related DEPS in AML

To characterize sex- and age-specific gene expression changes in AML patients compared to healthy individuals we conducted the following additional analyses detailed further below: (i) Analysis 2a: “Sex-relevance differential gene expression analysis and associated signaling pathways in AML”, and (ii) Analysis 2b: “Age-dependent differential gene expression analysis and associated signaling pathways in AML”. We used the same filtering criteria in both analyses as those used in Analysis 1 for identifying DEPS and signaling pathways between AML patients and healthy controls. In addition, DEPS were regarded as statistically significantly (up- or down-regulated) for each factor, sex and age, if they displayed p-value from Tukey’s HSD < 2.2 × 10^−7^ (Bonferroni^40^ adjusted p-value of 0.01 divided by the number of probe sets tested, 44,754).

#### Analysis 2a. Sex-relevance differential gene expression analysis and associated signaling pathways in AML

**Figure 3:**
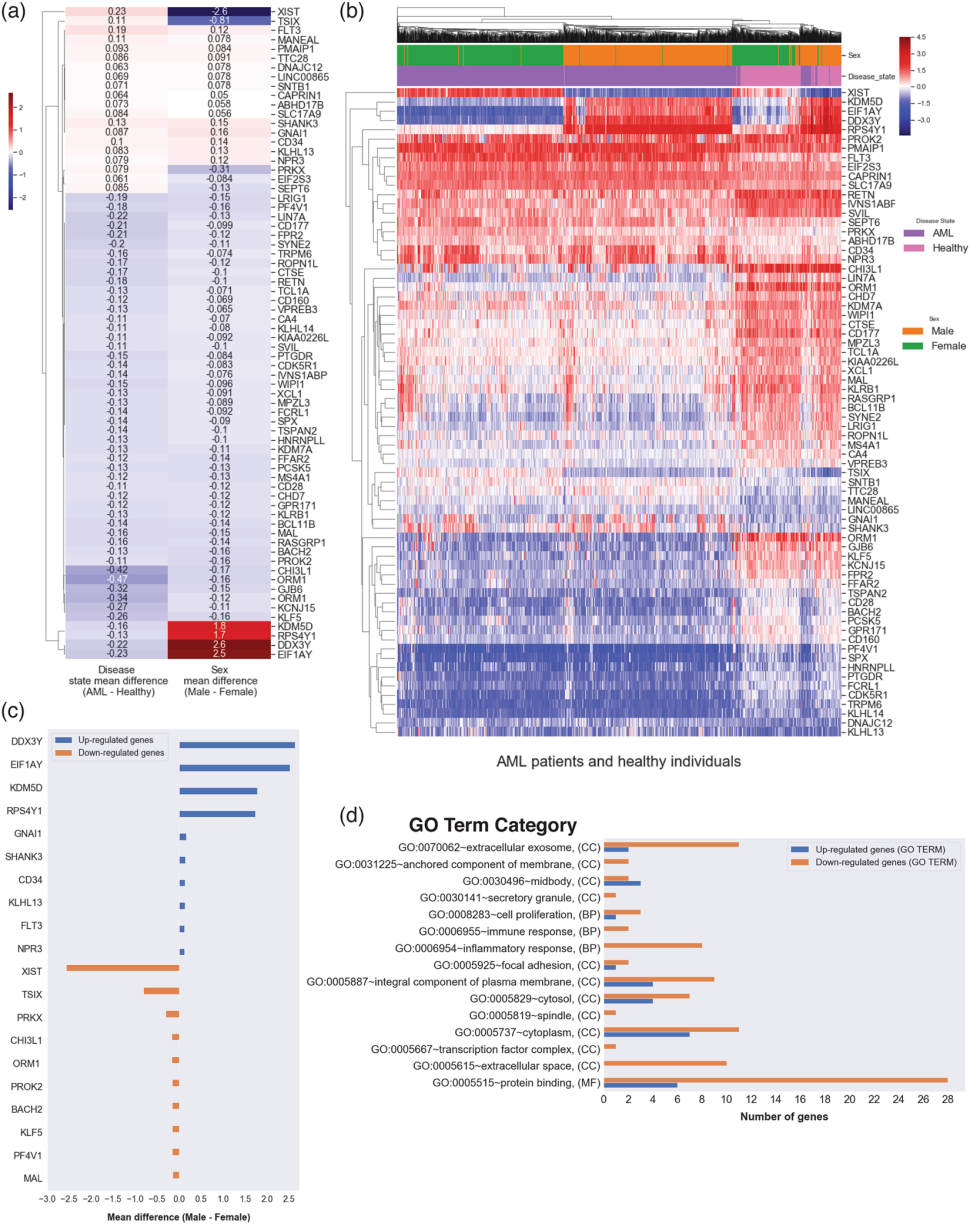
Sex-related gene expression analysis in AML. (**a**) The heatmap of mean difference values comparison between the 70 DE overlapping genes between Analysis 1 and Analysis 2a. (**b**) Heatmap the 70 DEPS expression (rows) on 2,761 arrays (columns) including 2213 AML patients and 548 healthy individuals from Analysis 2a of sex-relevance in AML (using unsupervised hierarchical clustering and Euclidean distance for clustering). The disease state (AML vs healthy) and sex of each subject are indicated in color bars at the top. (**c**) Horizontal bar plot of the top 10 DEPS (gene symbols on vertical axis), with the mean difference values between male-female (horizontal axis). (**d**) Enrichment analysis for statistically significant overrepresented biological GO terms on the 70 DE genes.

We identified 266 DEPS that show sex differences between AML patients (p-value < 2.2 × 10^−7^), as listed in Supplementary Table S4. 70 DEPS were found to overlap between Analysis 1 (AML disease state) and Analysis 2a (Sex-relevance in AML). Figure 3a shows these 70 DEPS with gene symbol annotations, and their mean difference values in the heatmap, which highlights differences in significance for common DEPS in both Analyses 1 and 2a. Figure 3b shows the hierarchical clustering of the 70 DEPS (rows) on sex and disease state of all 2,213 AML and 548 healthy subjects (columns) indicated by color bars above the heatmap. The top 10 DEPS higher in either males or females from this analysis are shown in Fig. 3c.

For enrichment analysis, we searched for common intersections in KEGG pathways and GO terms between the sex analysis and the 974 DEPS from the disease state analysis. Sex-relevant DEPS were found in 3 different signaling pathways, including genes higher expressed in males: FLT3 and CD34 in Hematopoietic cell lineage, FLT3 in Transcriptional misregulation in cancer 1, and PMAIP1 in p53 signaling pathway 1. MS4A1 was higher in females and found in the Hematopoietic cell lineage pathway (Table 3). Figure 3d shows GO analysis results, where 15 overrepresented biological GO terms were overlapped, including terms for extracellular space, immune response, protein binding, spindle, and midbody.

#### Analysis 2b. Age-dependent differential gene expression analysis and associated signaling pathways in AML

**Figure 4:**
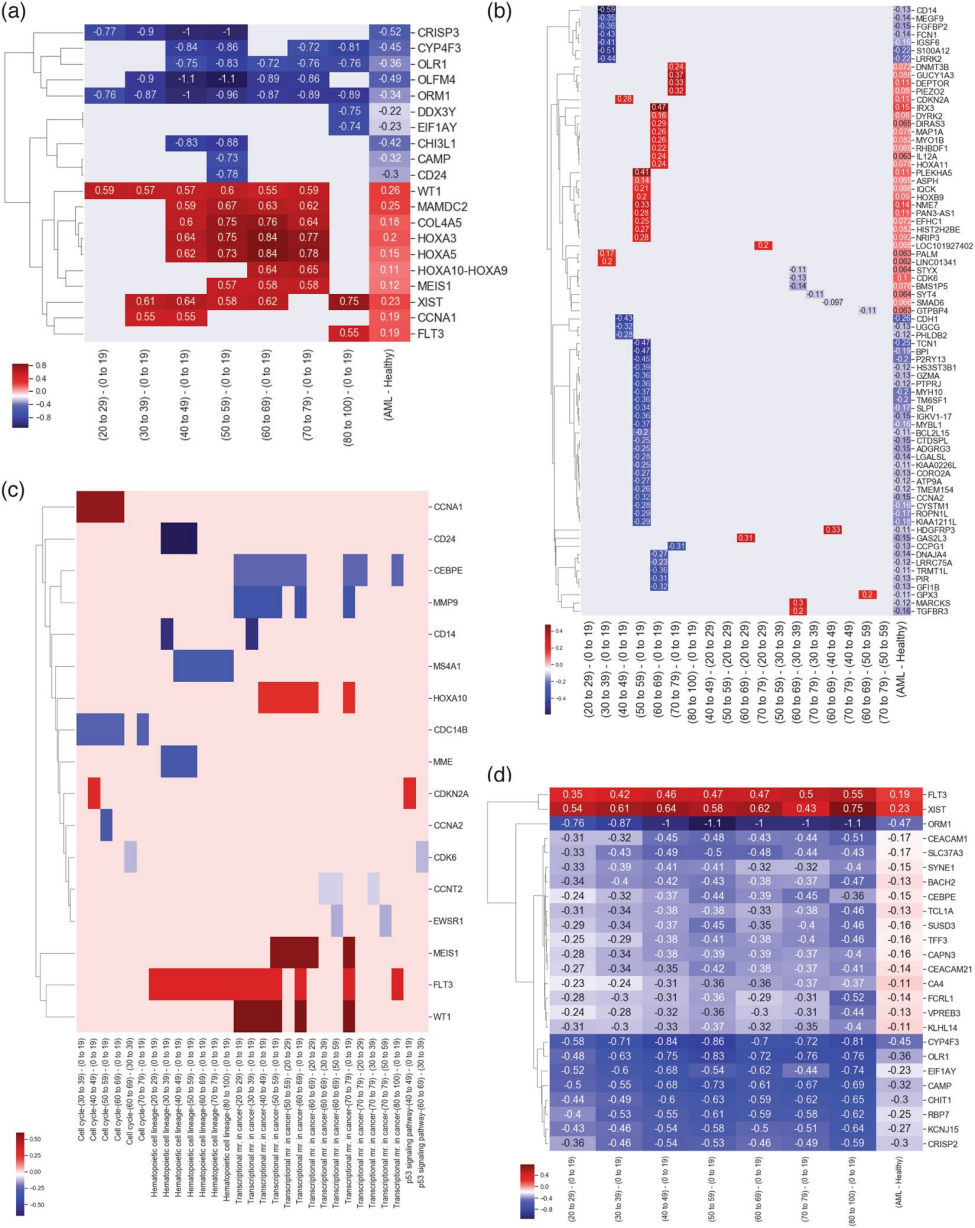
Age-related gene expression analysis in AML. (**a**) The top 10 up- and down- regulated DEPS overlapping AML and age-related analyses. (**b**) 75 DEPS specific to a single age-group comparison. (**c**) Overlaps over KEGG pathways of 17 DE genes identified in 4 KEGG pathways according to age groups. (**d**) The mean difference of 25 DEPS with respect to the 0–19 baseline across all other groups are plotted to illustrate changes with aging. The mean difference values between AML and healthy cohorts are shown in the right-most column of panes (**a**,**b** and **d**) for reference comparisons.

The subjects were binned in 8 age-groups: 0–19, 20–29, 30–39, 40–49, 50–59, 60–69, 70–79, and 80–100 years old. From this analysis, 1395 unique probe sets across all age-groups had statistically significant differential expression (p-value < 2.2 × 10^−7^, Supplementary Table S5). From these 375 unique DEPS (372 unique gene symbols) were found to overlap with the 974 DEPS probe sets from our AML disease state Analysis 1, accounting for an overall 1400 binary comparisons between the multiple age groups deemed statistically significant, based on Tukey HSD tests between age-group pairs. All 1400 identified pairwise differences between age groups and associated probe set/gene information can be found as Supplementary Table S6. The top 10 up- and down- regulated DEPS (labeled with gene symbols) from this analysis are shown in Fig. 4a. Additionally, 75 DEPS with gene symbols identified to have appeared specifically in one age-group comparison are shown in Fig. 4b. Through comparison with the results for KEGG analysis for signaling pathways from Analysis 1, 17 DE genes identified in all 4 KEGG pathways according to age groups (Fig. 4c, Table 4).

**Table 4:** KEGG pathway analysis of DEPS from analysis of 34 gene expression datasets overlap with age-specific findings.

AML age-dependent (AML - healthy) DEPS & associated signaling pathways			
Pathway	No. of genes*	Down-regulated Age-group	Up-regulated Age-group
**Hematopoietic cell lineage**	4, 1	CD14 (30 to 39)–(0 to 19)	FLT3 (20 to 29)–(0 to 19), (30 to 39)–(0 to 19), (40 to 49)–(0 to 19), (50 to 59)–(0 to 19), (60 to 69)–(0 to 19), (70 to 79)–(0 to 19), (80 to 100)–(0 to 19)
		MME (30 to 39)–(0 to 19), (40 to 49)–(0 to 19), (50 to 59)–(0 to 19)	
		CD24 (30 to 39)–(0 to 19), (40 to 49)–(0 to 19), (50 to 59)–(0 to 19)	
		MS4A1 (40 to 49)–(0 to 19), (50 to 59)–(0 to 19), (60 to 69)–(0 to 19), (70 to 79)–(0 to 19), (80 to 100)–(0 to 19)	
**Cell cycle**	3, 2	CCNA2 (50 to 59)–(0 to 19)	CCNA1 (30 to 39)–(0 to 19), (40 to 49)–(0 to 19), (50 to 59)–(0 to 19), (60 to 69)–(0 to 19)
		CDK6 (60 to 69)–(30 to 39)	
		CDC14B (30 to 39)–(0 to 19), (40 to 49)–(0 to 19), (50 to 59)–(0 to 19), (60 to 69)–(0 to 19), (70 to 79)–(0 to 19)	CDKN2A (40 to 49)–(0 to 19)
**p53 signaling pathway**	1, 1	CDK6 (60 to 69)–(30 to 39)	CDKN2A (40 to 49)–(0 to 19)
**Transcriptional misregulation in cancer**	5, 4	CD14 (30 to 39)–(0 to 19)	MEIS1 (50 to 59)–(0 to 19), (50 to 59)–(20 to 29), (60 to 69)–(0 to 19), (60 to 69)–(20 to 29), (70 to 79)–(0 to 19)
		MMP9 (20 to 29)–(0 to 19), (30 to 39)–(0 to 19), (40 to 49)–(0 to 19), (50 to 59)–(0 to 19), (60 to 69)–(0 to 19), (70 to 79)–(0 to 19)	
		EWSR1 (60 to 69)–(50 to 59), (70 to 79)–(50 to 59)	WT1 (20 to 29)–(0 to 19), (30 to 39)–(0 to 19), (40 to 49)–(0 to 19), (50 to 59)–(0 to 19), (60 to 69)–(0 to 19), (70 to 79)–(0 to 19)
		CEBPE (20 to 29)–(0 to 19), (30 to 39)–(0 to 19), (40 to 49)–(0 to 19), (50 to 59)–(0 to 19), (50 to 59)–(20 to 29), (60 to 69)–(0 to19), (70 to 79)–(0 to 19), (70 to 79)–(20 to29), (80 to 100)–(0 to 19)	FLT3 (20 to 29)–(0 to 19), (30 to 39)–(0 to 19), (40 to 49)–(0 to 19), (50 to 59)–(0 to 19), (60 to 69)–(0 to 19), (70 to 79)–(0 to 19), (80 to 100)–(0 to 19)
		CCNT2 (60 to 69)–(30 to 39), (70 to 79)–(30 to 39), (60 to 69)–(50 to 59)	HOXA10 (40 to 49)–(0 to 19), (50 to 59)–(0 to 19), (50 to 59)–(20 to 29), (60 to 69)–(0 to 19), (60 to 69)–(20 to 29), (70 to 79)–(0 to 19)

Enrichment analysis was done using 974 DEPS overlapped with age-specific analysis.

*Up and down regulated genes displayed.

To investigate further the progression with age, pairwise correlations between age-groups were computed. The 0–19 age-group was used as a common comparison reference with respect to other groups. Using this 0–19 group as a baseline, the mean differences of 25 DEPS with respect to the 0–19 baseline across all other groups were calculated and visualized in Fig. 4d. The mean difference values between AML and healthy are shown in the right-most column of Fig. 4a,b,d for reference.

We also wanted to assess the interaction of age with disease state. We filtered the 375 unique DEPS which intersected between the age and disease statistically significant DEPS, to also have a statistically significant interaction based on the ANOVA results (p-value < 0.01). This resulted in 43 unique DEPS, statistically significant for age, and for disease, and for interaction between age and disease in the linear model (Supplementary Table S6).

### AML classification machine learning model

We used the 974 DEPS from Analysis 1 to train a k-nearest neighbor (KNN) algorithm in ClassificaIO^41^. All 34 datasets (16 AML and 18 healthy) were used for training, and testing was performed on the 5 covariate reference datasets, which included both AML and healthy subjects (Table 1). The trained KNN algorithm was 97.9 ± 3% accurate, and 92% accurate in testing results (see Online Methods for parameters, Supplementary Table S1 and Fig. S4).

We also identified a minimum DEPS set that can have good predictive power and sensitivity: We first sorted the 974 disease-related DEPS based on the absolute value of their effect size (mean difference between AML and healthy patients). We then iteratively trained and tested a KNN model on the top *n* DEPS post sorting (Supplementary Fig. S5), incrementing *n* by one in each iteration. Based on the results, we picked the top 10 effect-sorted DEPS as a minimum set, as the graphs showed stabilization/saturation, with no substantial increase in performance after *n* = 10. The KNN model using these 10 effect-sorted DEPS had 96.1 ± 4% accuracy in training, and 90.9% accuracy in testing. (Supplementary Table S1, Fig. S5). The top 10 effect-sorted DEPS corresponded to the 10 top downregulated DEPS listed in Table 2.

## Discussion

In the present study, we reanalyzed data aggregated from our curation of 34 publicly available microarray gene expression datasets covering 2,213 AML patients and 548 healthy individuals to identify changes in AML gene expression associated with disease state (AML compared to healthy), sex-linked (male compared to female), and age-dependent (across age-groups compared to baseline). We performed 3 differential probe set (gene) expression and gene enrichment analyses, as discussed below.

### Analysis 1

*Gene expression analysis and associated signaling pathways of AML disease state compared to healthy individuals*, was carried out to identify DEPS in AML disease state. The results from this analysis were then used as a baseline indicator for AML disease state. 974 DEPS (487 up- and 487 down-regulated) were identified as statistically significant differentially expressed between AML patients and healthy individuals (p-value < 0.01) and showing high effect size (5% 2-tailed quantile selection). Among these, 6 genes are known to be involved in AML functional pathways, including 4 up-regulated, JUP, CCNA1, FLT3, PIK3R1, and 2 down-regulated, CD14, CEBPE. The top 10 up- and down-regulated genes from this analysis are listed in Table 2. As shown in Fig. 2b of the top 10 up- and down-regulated DEPS and corresponding gene annotations, WT1 (Wilms tumor 1) was found to be the most expressed and CRISP3 (cysteine-rich secretory protein 3) was the most under-expressed gene. WT1 is a transcriptional regulatory protein essential for cellular development and cell survival, and it has been shown to be highly expressed with an oncogenic role in AML^42,43^, in agreement with our findings. However, CRISP3’s direct role in AML is still under investigation. CRISP3 is a member of the cysteine-rich secretory protein CRISP family with major role in female and male reproductive tract, and is mainly expressed in salivary glands and bone marrow^44^. Recently, 80 genes were reported as “extracellular matrix specific genes” in leukemia, and CRISP3 was among the downregulated DE genes reported^45^. CRISP3 associations with AML merit further investigation.

The enrichment analysis for GO terms of the 974 DE probe sets (Fig. 2c) resulted in 727 identifiers (335 up- and 392 down-regulated) enriched for 21 GO terms. 592 of these (257 up- and 335 down-regulated) were enriched in the cellular component (CC) categories mainly associated with cytoplasm, extracellular exosome, cytosol, and extracellular space. These terms are rather generic, but may still reflect relevance to AML development and progression^46,47^. GO terms in the Biological process (BP) category included inflammatory and immune responses, and cell proliferation, which are expected as AML is characterized by terminal differentiation of normal blood cells, and excessive proliferation and release of abnormally differentiated myeloid cells, which affects many biological processes associated with the immune system. The four statistically significant KEGG pathways identified in the pathway enrichment analysis encompassed 56 DEPS (Table 3). Transcriptional misregulation in cancer was the most up-regulated pathway in AML (13 up-regulated DE genes), while Hematopoietic cell lineage, and Cell cycle pathways were mostly down-regulated, and the p53 signaling pathway was balanced in terms of up/downregulated DE genes (Fig. 2c). For the enriched pathways, Fig. 2d shows the mean difference values of the 56 DE pathway-associated genes, including 27 up- and 29 down- regulated genes. These KEGG pathways are known to be involved in tumorigenesis. Additionally, the majority of the DE genes from the AML analysis associated with the identified signaling pathways are known to be abnormally expressed in AML. These findings are consistent with results from other studies, and our current understanding of AML pathogenesis.

The DEPS overlap with the 25 genes reported by Miller and Stamatoyannopoulos that were reported in at least 8 studies^25^, namely HOXA10, CD34, MEIS1, VCAN, RBPMS and MN1. In terms of the genes reported in the same study for poor progression we also consistently identified as upregulated HOXA10, RBPMS, CD34, GNAI1, CLIP2, DAPK1, GUCY1A3, ANGPT1 and FLT3, and as downregulated UGCG. While these are known markers, with consistent expression differences, our additional results need to be investigated further and experimentally validated, including mechanistic considerations.

### Analysis 2a

*Sex-dependent gene expression analysis and associated signaling pathways in AML compared to healthy individuals*, was performed to explore the relevance of patients’ sex on gene expression and to identify sex-linked genes and associated signaling pathways in AML. A total of 266 DEPS were found statistically significant in this analysis, with 70 found to overlap with the DEPS from Analysis 1 (Fig. 3a,b). The top 10 up- and down-regulated DE genes with respect to females include (Fig. 3c): (i) DDX3Y (DEAD-Box Helicase 3 Y-Linked), EIF1AY (Eukaryotic Translation Initiation Factor 1 A Y-Linked), KDM5D (Lysine Demethylase 5D), RPS4Y1 (Ribosomal Protein S4 Y-Linked 1) with higher expression in males compared to females, and (ii) XIST (X Inactive Specific Transcript), TSIX (TSIX Transcript, XIST Antisense RNA), and PRKX (Protein Kinase X-Linked) with higher expression in females. These genes are known to be sex-specific and show expression differences and sex separation within the AML and the healthy groups respectively (Fig. 3d). The role of these genes as positive controls in studies with AML needs to be investigated further. We also reported sex and AML known genes that were statistically significant in our analysis, including FLT3 and MAL.

### Analysis 2b

*Age-dependent gene expression analysis and associated signaling pathways in AML compared to healthy individuals*, was carried out to identify common set of age-dependent gene expression and associated signaling pathways and to explore age-dependent trends in AML. The age-dependent analysis using ANOVA, identified 1,395 DEPS (p-value < 2.2 × 10^−7^). To identify age-related DEPS in AML we overlapped the 1,395 DEPS to our findings of 974 DEPS in AML disease state (Analysis 1) (Fig. 4), and identified an overlap of 375 DEPS (p-value < 2.2 × 10^−7^). The top 10 up and down DE age-associate genes in AML according to the mean difference values in seven age-groups are shown in Fig. 4a (including their corresponding values from AML disease state in column “AML - healthy” for comparisons). Interestingly, CRISP3 was among the down regulated genes in this analysis as well, specifically associated with differences in younger age groups, 20 to 49 years of age as compared to the 0 to 19 age group. Other genes showing age-specific differences included HOXA3, HOXA5 and HOXA10-HOXA9, which belong to the homeobox genes (HOX) family of transcription factors, essential for embryonic development and hematopoiesis, and associated with chromosomal abnormalities translocation and over-expression in AML^48,49^. Also identified with age-specific DE, was ORM1, which in Analysis 1 was among the top-10 under-expressed genes, and was also among the 70 DE genes in analysis 2a. ORM1’s direct role in AML also merits further investigation, given ORM1 involvement in immunosuppression and inflammation^50^. Finally, we have identified 75 DEPS that show association with only one age-group, exclusively from all other age-groups, suggestive of potential age-specific differential gene expression signature.

We further wanted to assess the interaction of age with disease state. From the 375 unique DEPS which intersected between the age and disease analysis, 43 unique DEPS showed statistically significant interaction between age and disease in the linear model (p < 0.01, Supplementary Table S6). Among the 43 DEPS are 13 immune disease related genes^37,38^ such as SOCS5 and SOCS6 (suppressors of cytokine signaling, whose role in cancer is still under investigation^51^), EBF1 (early B-cell Factor 1), CD160, TCL1A (T-cell leukemia/lymphoma 1A), VPREB3 (pre-B lymphocyte 3), KLF10 (Kruppel-like factor 10), NTM (neurotrimin), PLXNA4 (plexin A4), SLC25A21, SYT4 (synaptotagmin 4) and TCERG1 (transcription elongation regulator 1). While these genes/gene families have been associated with cancer^13,52^, their potential role in AML is still under and merits further investigation. These 43 DEPS with statistically significant age-disease interactions may be important in AML development, particularly for detecting early markers of AML, potentially identifying preleukemic conditions, and using these markers as treatment targets.

### Disease status classification

Using the differential expression analysis results combined with various machine learning models, we were able to identify gene expression signatures for AML that we used for training a predictive KNN model of health status (AML/healthy) with 96.1 ± 4% training accuracy. The model uses a minimal set of 10 DEPS (determined through iteration using an increasing number of DEPS ranked by their absolute effect difference (mean differences between AML and healthy – see Online Methods and Results). The feature set coincides with the top 10 down-regulated DEPS for Analysis 1 (disease), Table 2. The trained KNN model was tested on the 5 covariate datasets, with high performance: 90% accuracy, 92.97% specificity, 84.81% sensitivity, 80.7% precision, and a receiver operating characteristic (ROC) area under the curver (AUC) of 88.89 (Supplementary Fig. S5). The set of genes can have a diagnostic impact, but will need to be validated experimentally, and additionally in same-tissue cohorts.

Our study identified multiple potentially significant DEPS, with age and sex related differences associated with AML. While our findings may generate further hypothesis-driven investigations, we need to also identify the study’s limitations: The primary limitation is that the analysis of AML and healthy subjects involved bone-marrow and blood samples respectively in each disease group. We tried to account for this utilizing tissue as an effect in our linear model, and including multiple interactions. Other limitations include an unbalanced AML/healthy ratio, as well as the lack of in-study healthy controls. To address these we attempted to account for batch effects using a dataset-wise iterative batch correction transformation, as discussed in the methods. Finally, we also included binary interactions between the factors in the analysis to account for interaction-related confounding effects. Additionally, the study is limited by the available data, particularly for prognostic utility. The low numbers of PB data, as well as the lack of healthy BM data do not allow for an equal-footing comparison of differences in AML between PB and BM cell gene expression signatures. Furthermore, the prognostic utility of the study is limited by the lack of uniformly reported or sparse clinical data, including progression/relapse-free survival, (in-)effective therapeutic intervention, such as bone marrow transplantation or pharmaceutical treatment, or mutational status. More uniform reporting of published metadata would greatly improve the utility of publicly available datasets. Additionally, more data would be necessary to fully evaluate mutational status and AML classifications. We anticipate that the use of sequencing data now being generated (e.g. RNA-sequencing data) as well as single cell level gene expression, will allow better concurrent determination of mutational status, global gene expression, and cell-type specific evaluation of patient samples.

In summary, our study successfully integrated multiple datasets to perform an analysis of gene expression in AML, across multiple factors that included disease, sex and age considerations, and identified interesting genes, both known and not previously reported as differentially expressed in each factor. We identified 974 DEPS and 4 associated significant pathways involved in AML, and 70 sex- and 375 age-related DE signatures. Using the 10 of the 974 DEPS, a KNN model allowed AML classification with 90.9% accuracy. We hope that these findings may provide additional relevant targets for further experimental mechanistic studies, and to help identify new markers and therapeutic targets for AML.

## Methods

The generalized workflow consisted of five main steps: i) Curation of microarray gene expression data, ii) Preprocessing of raw data files followed by batch effect correction, iii) Predictions of missing annotations using supervised machine learning, iv) Differential gene expression analysis, and v) Pathway analysis, that included gene annotation, and finally gene expression-based prediction of AML (Fig. 1a).

### Gene expression data curation and screening criteria

The datasets used in this study were selected from the GEO public repository, maintained by the National Center for Biotechnology Information (NCBI)^53^ (https://www.ncbi.nlm.nih.gov/geo/). To facilitate speed of search and keep up-to-date with possible new and relevant datasets, as soon as they were released, a Python script was used that utilized functions from the Entrez Utilities from Biopython^54^. We used the script to navigate the GEO records, and downloaded microarray gene expression datasets up to October 2018. We additionally utilized Python packages, including Pandas, NumPy, and Matplotlib for data structure, numerical computing for data processing, and data visualization respectively. We used strict inclusion criteria to maintain consistency in each dataset selection, screening for availability of both raw and data annotation files provided, human samples used from untreated subjects, and that the sample source was from either BM or PB. Array platform was restricted to Affymetrix, which was found to have the most available data, and to avoid cross-platform normalization issues. Inclusion criteria and the data curation workflow are illustrated in Fig. 1a,b.

### Gene expression datasets used in our analysis

The curation method is summarized in the Supplementary File S3 flowchart and in the Results section. For our analysis we included 34 age-dependent datasets from 32 different studies, 16 included AML and 18 healthy subjects respectively. From the 34 datasets, 32 were produced from Affymetrix GeneChip Human Genome U133 Plus 2.0 (GPL570) and 2 conducted on Affymetrix GeneChip Human Genome U133 Array Set (GPL96 & GPL97) arrays. Table 1 provides detailed information about each dataset, including the number of samples used from each dataset, sample tissue source, as well as the total number of AML patients and healthy subjects. Two studies, GSE12417^55^ and GSE37642^56–59^, were originally conducted on two different Affymetrix array types (GPL570, and GPL96 & GPL97), so each was separated into two subgroups and each subgroup was considered as individual dataset in our analysis, dataset GSE12417: (i) subgroup 1 included 73 BM and 5 PB samples, and (ii) subgroup 2 included 160 BM and 2 PB. For dataset GSE37642: (i) subgroup 1 included 140 BM and (ii) subgroup 2 included 422 BM samples (Table 1).

### Dataset annotation and pre-processing

Figure 1b outlines the workflow of our preliminary data analysis including pre-processing. For each dataset used in our analysis, raw microarray CEL files were downloaded from GEO, metadata was reviewed, and the data was manually curated to guarantee that each array corresponded to either an AML patient or healthy individual, was verified as correctly annotated for sample source (BM or PB), platform technology used, age, sex, and disease state (AML or healthy). For each individual dataset, raw CEL files were grouped and pre-processed together using the RMA (Robust Multi-Array Average) algorithm^60–62^. Datasets with mixed sample source, i.e. both BM and PB, were pre-processed together irrespective of sample source. Pre-processing consisted of: correction for background noise using RMA background correction on perfect match (PM) raw intensities; quantile normalization to obtain the same empirical distribution of intensities for each array; median polish summarization of probes into probe sets to estimate gene-level expression value: and logarithm base-2 transformations of gene expression values to facilitate data interpretation (normal distributions) and comparisons between arrays. Additionally, the expression data were reduced to 44,754 probe sets that overlapped across all datasets. Finally, within each dataset we standardized across all probe sets, by subtracting the mean and dividing by the standard deviation to obtain a Z-score.

### Prediction of missing sex and sample source annotations from curated datasets

From the curated datasets, 805 arrays (802 AML patients and 3 healthy subjects) and 737 arrays (all AML patients) were missing sex or sample source annotations respectively. Without these metadata, we would have to discard the data, which in turn would limit the statistical power for the study, and our ability to correct for biases stemming from individual datasets^26^. To address this, we used supervised machine learning classifiers to predict both sex and sample annotations. For all predictions, we used ClassificaIO^41^, a machine learning for classification graphical user interface, which we recently developed, that utilizes the scikit-learn machine learning package in Python^63^.

To predict sex in pre-processed datasets, 1956 arrays (including both healthy and AML), that included 44,754 probe sets and their annotated sex information, were used to train a logistic regression (LR) classification model, and to predict 805 sex annotations. Additionally, 2,024 arrays were used to train for sample source, with prediction performed on 737 arrays.

The supervised machine learning LR classifier we used had the following parameters (descriptions based on scikit-learn documentation^63^):

- *random_state* = *None*: specifies whether a seed should be used for the pseudorandom generator in selecting training and testing subsets.
- *shuffle* = *True*: determines that data will be shuffled before splitting for training and testing.
- *penalty* = *l2*: determines that an L2 norm should be used for penalization.
- *multi_class* = *ovr*: specifies that a binary problem is fit for each label.
- *solver* = *liblinear*: specifies that liblinear is the algorithm used in the optimization.
- *max_iter* = *100*: maximum number of iterations for the solvers to converge.
- *tol* = *0.000*1: tolerance for stopping criteria.
- *fit_intercept* = *True*: a constant (bias/intercept) is added to the decision function.
- *intercept_scaling* = *1.0*: a “synthetic” feature with constant value equal to intercept_scaling is appended to the instance vector.
- *Verbose* = *0*: turns off verbosity in evaluation.
- *n_jobs* = *1*: number of CPU cores used.
- *C* = *1.0*: inverse of regularization strength.
- *dual* = *False*: dual or primal formulation.
- *warm_start* = *False*: do not reuse the solution of the previous call to fit as initialization.
- *class_weight* = *None*: all classes assumed to have weight one.

Confusion matrix details, model accuracy and error for training and testing are presented in Supplementary Table S1, Fig. S1 and results in Supplementary Files S1 and S2. To account for training overfitting, we used 10-fold cross-validation on all 1,956 gene expression data arrays for training and validation, implemented automatically in scikit-learn.

### Dataset-wise correction approach for batch effects correction

Batch correction was done using a dataset-wise correction. Here we refer to the term “dataset-wise correction” to indicate performing batch correction iteratively on one dataset at a time, against a reference set of datasets chosen to account for variability. To account for the lack of within-study healthy controls in the curated gene expression datasets, we used 5 additional datasets that included within-study controls, with GEO accessions: GSE107968, GSE68172^64^, GSE17054^65^, GSE33223^66^, and GSE15061^67^ (Table 1). We refer to the latter datasets as “covariate” reference datasets, as they were used as the reference datasets in the batch correction. Our approach aimed to balance/distribute the weight of batch effects exerted by each dataset, as this is dependent on the number of observations within a given dataset. Combined, the covariate reference datasets included 613 total arrays (455 AML and 158 healthy controls). We used ComBat^27^ to correct for study batch effects, as its empirical Bayes-based algorithm uses both scale and mean center based methods, providing an appropriate algorithm^27^. Covariate reference datasets were treated as the covariate for batch during batch correction, to improve performance in correcting for batch effects rather than biological variation. After batch correction, we used principal component analysis (PCA), visualizing components in both 2 and 3 dimensions, to compare the clustering results for corrections (Supplementary Fig. S2). Covariate reference datasets were removed after the batch correction step and were not part of our downstream linear model analysis (as they lacked age annotations). The covariate datasets were used for testing of the AML prediction models discussed below.

### Gene expression linear model analysis

After the batch correction step, we performed differential gene expression analysis on the merged datasets (34 datasets, 16 AML and 18 healthy), using the expression values for all 44,754 common probe sets. The effects of patients’ age, sex, and sample source, including their pairwise interactions were investigated using an analysis of variance (ANOVA)^28,30^. For each probe set *i*, where *i* = [1, 2, … 44, 754], the expression *Y*_*i*_ was modeled with a linear model:

$${Y}_{i}\sim a+s+d+t+a\,:s+a\,:d+a\,:t+s\,:d+s\,:t+d\,:t+\varepsilon ,$$

where *d* is the disease state (AML or healthy), *a* is age (between 0 to 100 years), *s* is sex (female or male), *t* is sample source (BM or PB), and *ε* is a random error term, and colons represent interactions between factors. We note that the model includes sample source and its interactions to address comparisons involving different tissues in AML and healthy subjects (BM or PB respectively). The selection of using a linear model was based on having multiple factors to capture in the analysis, and also having a large number of samples (by integrating multiple datasets) – in that the Central Limit theorem allows for the assumptions for F-test to hold for ANOVA. We also evaluated fit residuals’ distribution for normality by plotting Quantile-Quantile (QQ) plots and density distributions.

Based on the ANOVA we first identified statistically significant differences for the disease state factor (p-value < 0.01). To identify statistically significant level differences (between AML and healthy) we then carried out post-hoc analyses for each statistically significant probe set using Tukey’s HSD tests implemented in R, (selecting probe sets with Tukey HSD p-value < 0.01). Finally, to focus on biological effects, we filtered the results to have mean difference values (i.e. differences between the means of AML and healthy groups) in the <5% and/or >95% quantiles of the overall mean difference distribution across probe sets. The final set of the results are referred to as differentially expressed probesets (DEPS) with respect to the disease.

### Pathway enrichment analysis and functional annotation

We carried out enrichment analysis (overrepresentation) for DEPS using the database DAVID^37,38^ for KEGG signaling pathways^32–34^ and GO functional annotation terms^35,36^. Pathways and terms identified were deemed statistically significant based on Benjamini-Hochberg adjusted p-value < 0.05.

### Using a k nearest neighbor model to predict AML

To predict AML health status, normalized intensities from DEPS (with respect to disease) were used as features for training a k-nearest neighbor (KNN) model (implemented in ClassificaIO^41^). All 34 datasets (16 AML and 18 healthy) were used as training data. Testing of the model was done independently of training on all 5 covariate datasets. The KNN model used the following parameters (please refer to scikit-learn documentation for further details^63^):

- *random_state* = *None*: specifies whether a seed should be used for the pseudorandom generator in selecting training and testing subsets.
- *shuffle* = *True*: determines whether or not data will be shuffled before splitting for training and testing.
- *metric* = *minkowski and p* = 2: define which metric to use. The *minkowski* metric is using the Minkowski distance of order *p* between two *n*-dimensional vectors $X=\{{x}_{1},{x}_{2},\,\ldots \,{x}_{n}\},$ and $Y=\{{y}_{1},{y}_{2},\,\ldots \,{y}_{n}\}$, which is defined as $d(X,Y)=(\sum _{i}\,{|{x}_{i}-{y}_{i}|}^{p}{)}^{\frac{1}{p}}$.
- *weights* = *uniform*, defines that uniform weights will be used so that all points in each neighborhood are weighted equally.
- *metric_params* = *None*: additional metric parameters (none used in this case).
- *algorithm* = *auto*: automatically determines the algorithm to use for computing nearest neighbors, can internally use a *BallTree* or *KDTree* or brute force algorithm.
- *n_neighbors* = 30: number of nearest neighbors to be used.
- *leaf_size* = 30: leaf size passed to *BallTree* or *KDTree* algorithms.
- *n_jobs* = 1: number of parallel jobs to run for neighbors search.

Details of training and testing are given in Supplementary Table S1 and Fig. S4.

To identify a minimum set of DEPS with good predictive power and sensitivity, we first ranked the 974 disease-related DEPS based on the absolute value of their effect size (mean difference between AML and healthy patients). We then iteratively trained and tested a KNN model on the top *n* DEPS (Supplementary Fig. S5), incrementing *n* by one in each iteration. Based on the results, we picked the top 10 effect-ranked DEPS as a minimum set, as the graphs showed stabilization/saturation, with no substantial increase in performance after *n* = 10. We then trained a KNN model using these 10 effect-sorted DEPS, using the same parameters as listed above (Supplementary Table S1, Fig. S5).

## Supplementary information

Supplementary Information

Supplementary Tables

Supplementary File 1

Supplementary File 2

Supplementary File 3

## Data Availability

The datasets generated in the study, supplementary data, tables, figures and files are available online at 10.5281/zenodo.3257786. Datasets re-analyzed in the study are publicly available on the Gene Expression Omnibus repository, at https://www.ncbi.nlm.nih.gov/geo/ under the accessions summarized in Table 1.
