# Supplementary material for: Multi-study reanalysis of 2,213 acute myeloid leukemia patients reveals age- and sex-dependent gene expression signatures
Source: Sci Rep. 2019 Aug 27;9:12413. doi: 10.1038/s41598-019-48872-0 (PMC6712049; doi:10.1038/s41598-019-48872-0)
Supplement: Supplementary file 1 — Supplementary Information [file 41598_2019_48872_MOESM1_ESM.pdf]

SUPPLEMENTARY INFORMATION

Multi-study reanalysis of 2213 acute myeloid leukemia patients reveals age- and sex-dependent gene expression signatures

Raeuf Roushangar [1, 2], George I. Mias [1, 2*]

[1] Department of Biochemistry and Molecular Biology,

[2] Institute for Quantitative Health Science and Engineering,

Michigan State University, East Lansing MI 48824, USA

*Corresponding author

E-mail: gmias@msu.edu (GM)

A. Supplementary Figures

Supplementary Figure S1: Classification of missing metadata annotations.

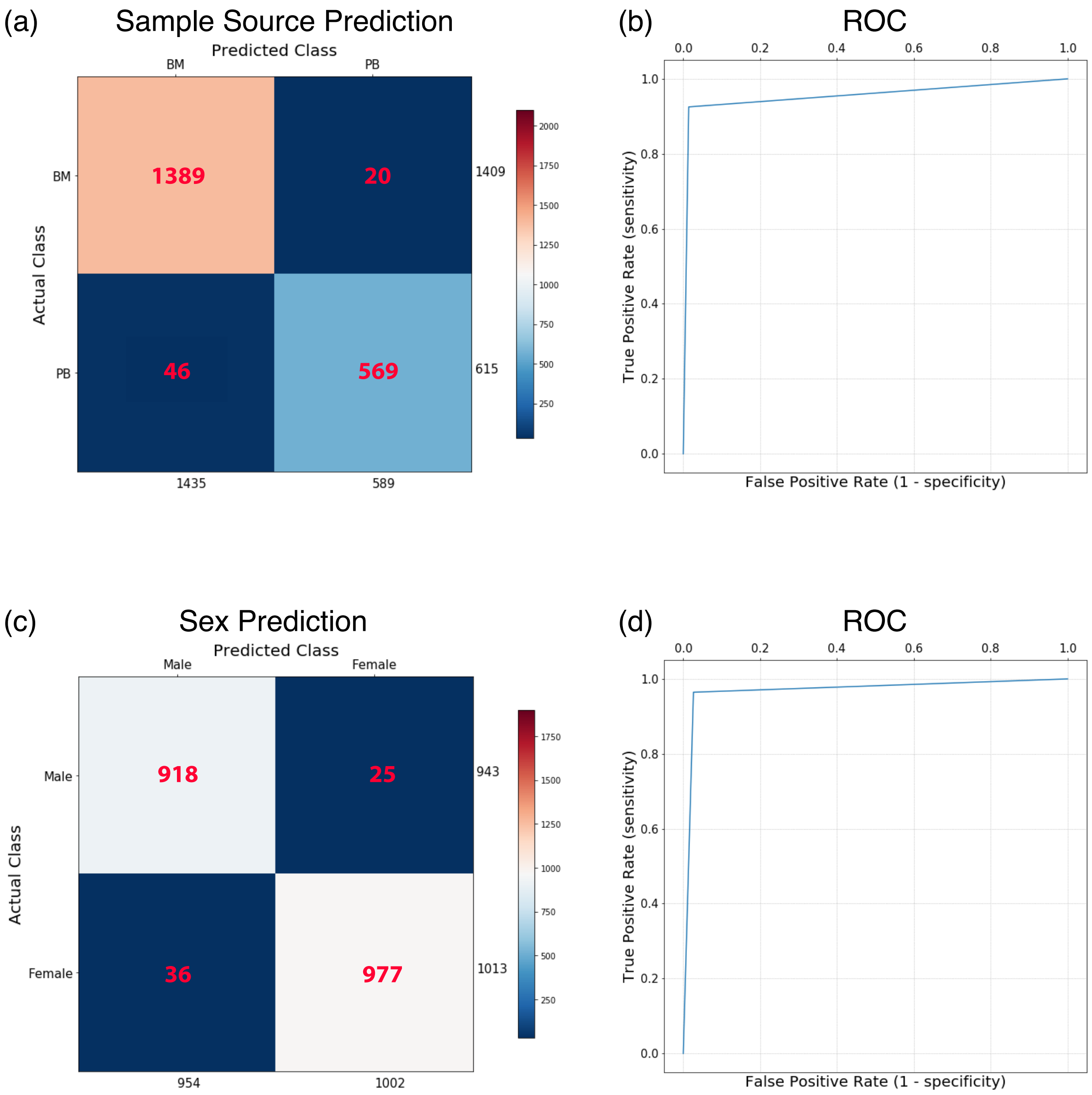

Classification of missing metadata annotations. 805 (802 AML and 3 healthy) of the 2,761 curated arrays were found to be missing sex annotation, and 737 arrays (all AML patients) were missing sample source annotations. A logistic regression (LR) classification model was used for prediction, with the training confusion matrix and receiver operating characteristic ROC curves shown above for sample source prediction (a-b) and sex prediction (c-d) respectively. See also Supplementary File S1 for model statistics.

Supplementary Figure S2: Principal component analysis.

(a)

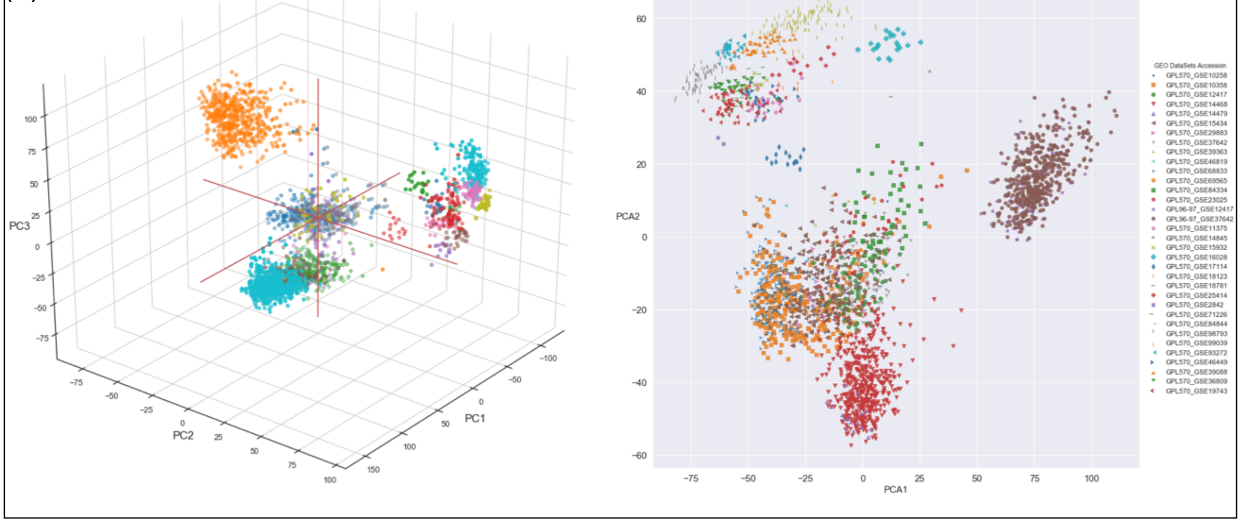

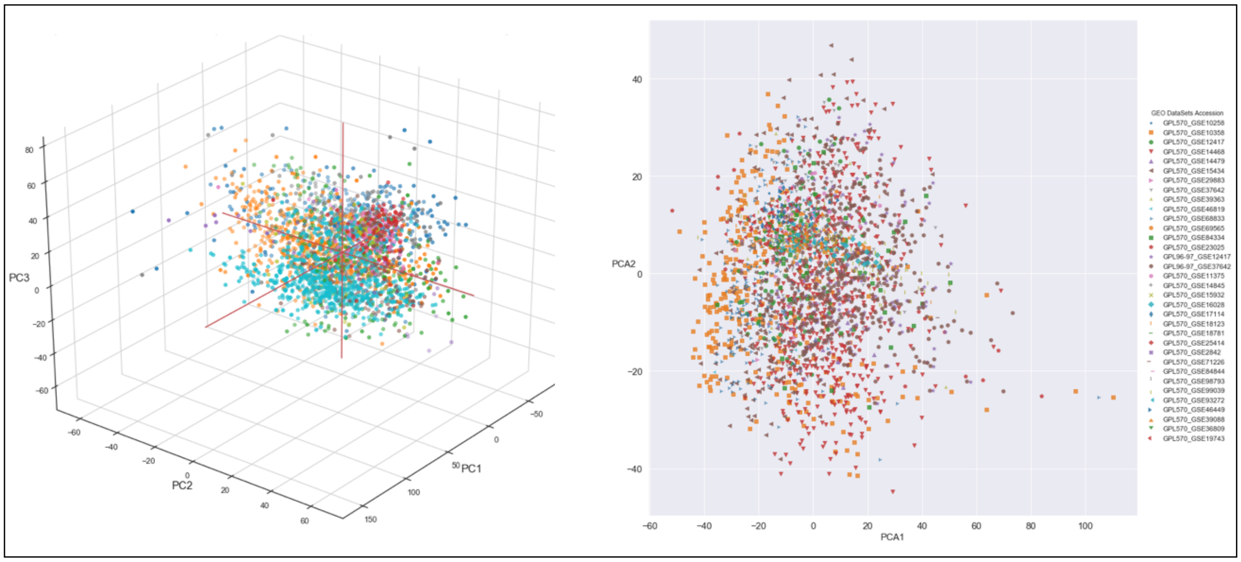

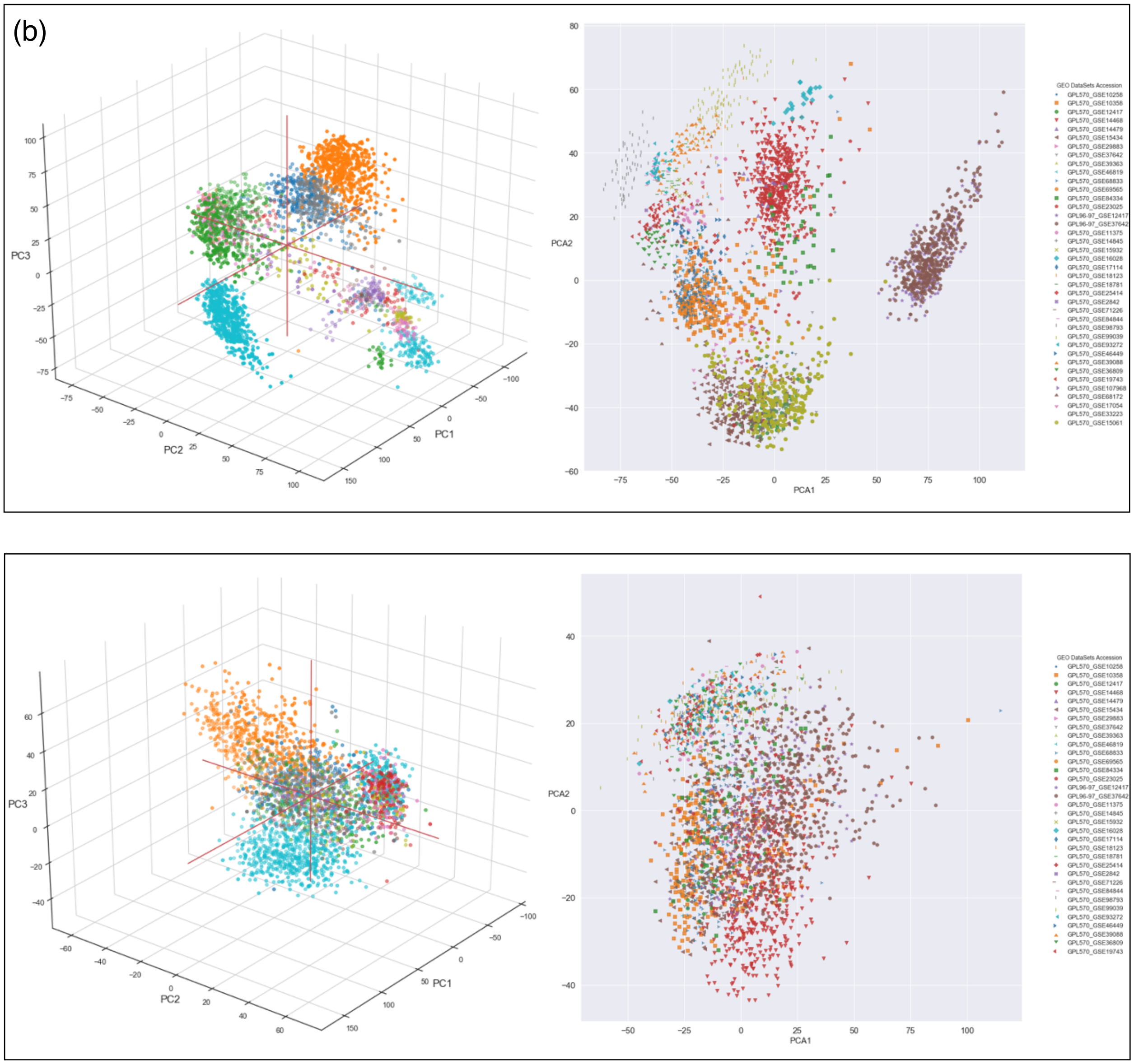

Principal Component Analysis (PCA). PCA of pre-processed, logarithm base-2 transformed, z-score standardized data sets with 44,754 probe sets across all data sets: **(a)** 2761 subjects (AML and healthy), and data before batch correction **(top)**. The data was corrected without covariates resulting to loss of biological effect information due to lack of within-sample controls **(bottom)**. **(b)** Data shown before batch effect correction including 2761 subjects (AML and healthy), and additionally 613 covariate datasets (5 data sets), (labeled green) **(top)**, and, **(bottom)** PCA of, data post batch effect correction using ComBat as described in the methods section.

Supplementary Figure S3: KEGG Pathways.

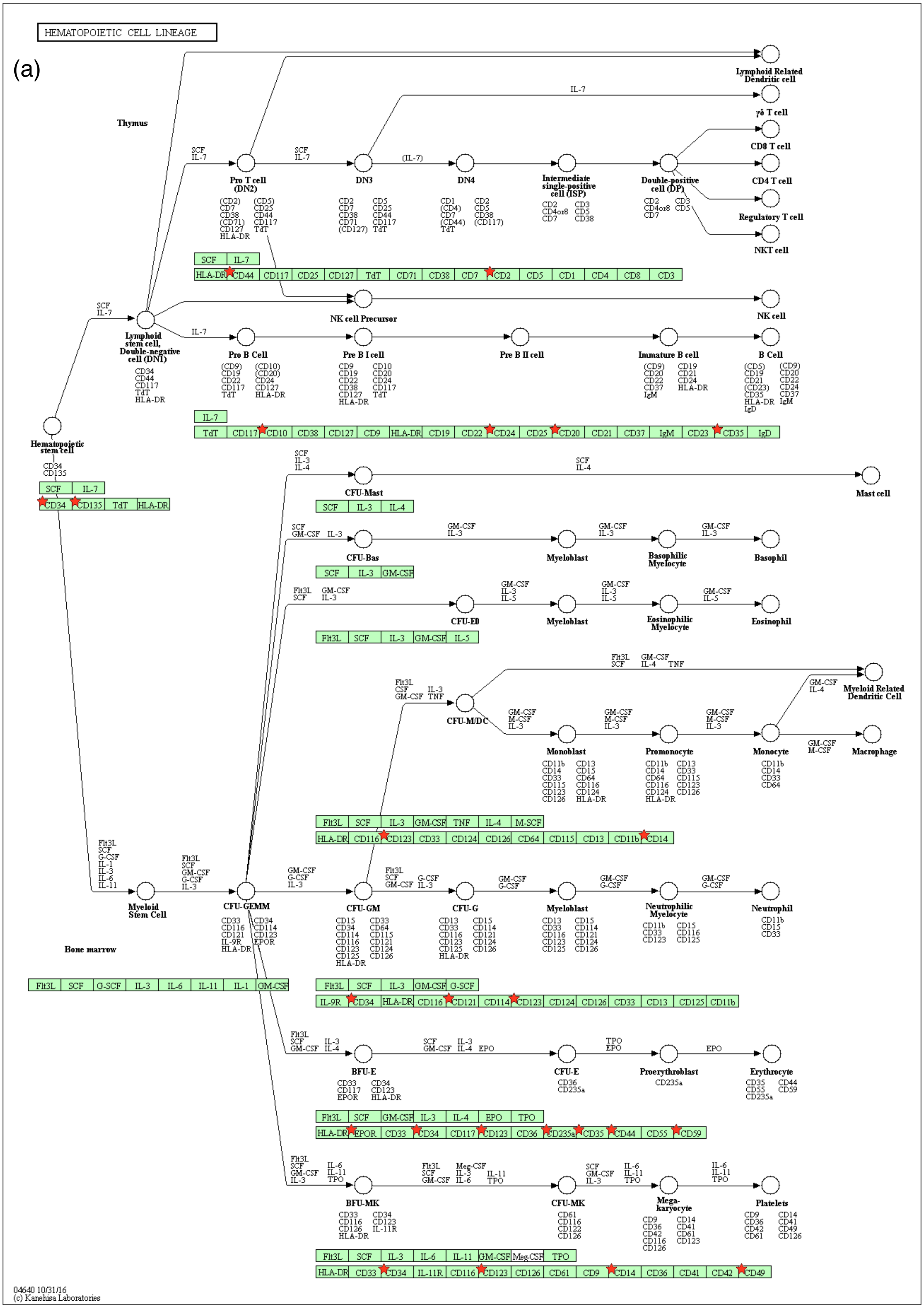

(b)

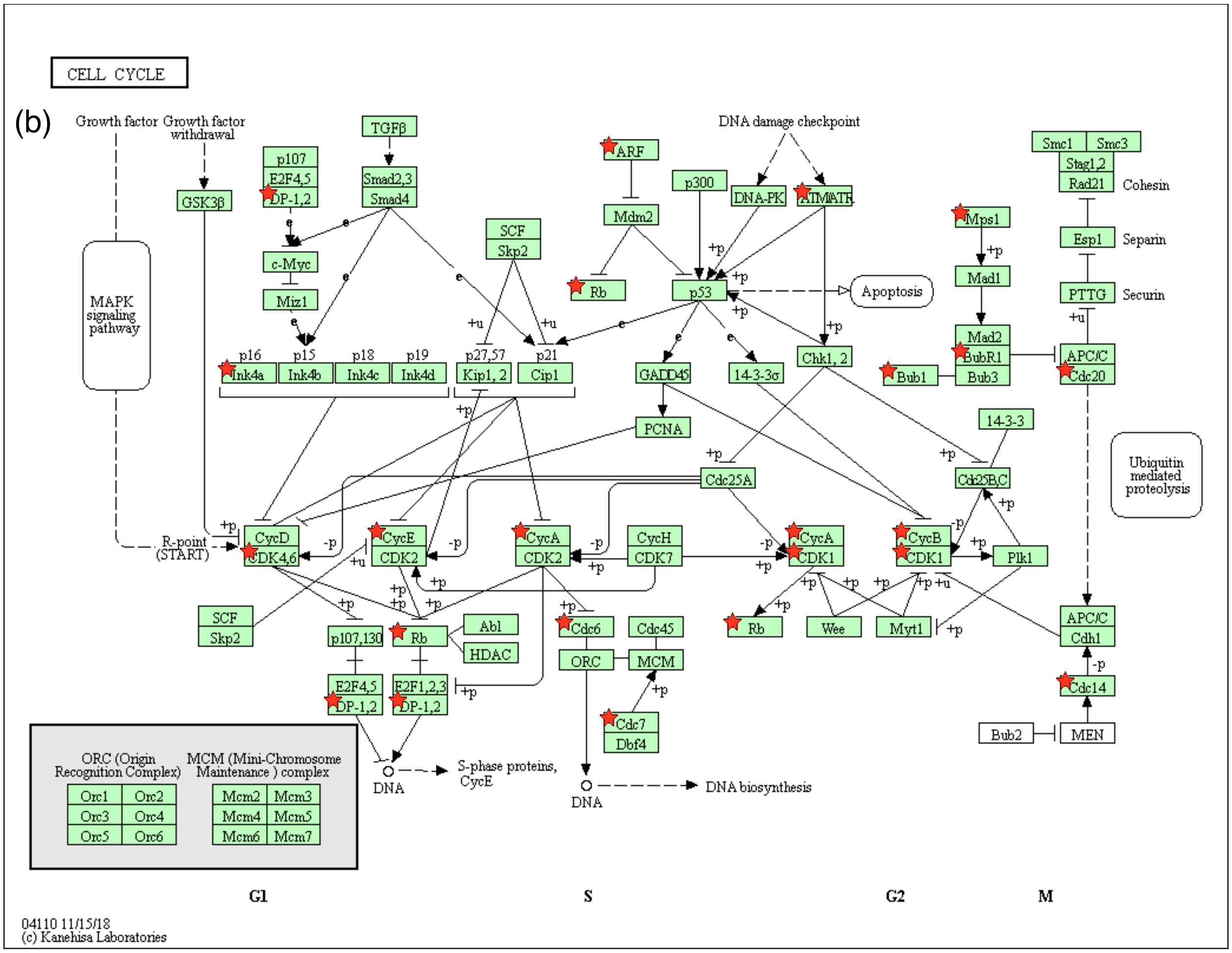
CELL CYCLE
Growth factor
Growth factor withdrawal
MAPK signaling pathway
GSK3β
p107
E2F4,5
DP-1,2
TGFβ
Smad2,3
Smad4
c-Myc
Miz1
SCF
Skp2
ARF
Mdm2
Rb
p300
p53
DNA damage checkpoint
DNA-PK
ATM/ATR
Apoptosis
Mps1
Mad1
Mad2
BubR1
Bub3
Bub1
Smc1
Smc3
Stag1,2
Rad21
Cohesin
Esp1
Separin
PTTG
Securin
APC/C
Cdc20
p16
p15
p18
p19
Ink4a
Ink4b
Ink4c
Ink4d
p27,57
p21
Kip1, 2
Cip1
GADD45
14-3-3σ
Chk1, 2
PCNA
14-3-3
Cdc25A
Cdc25B,C
Ubiquitin mediated proteolysis
R-point (START)
CycD
CDK4,6
CycE
CDK2
CycA
CDK2
CycH
CDK7
CycA
CDK1
CycB
CDK1
Plk1
SCF
Skp2
p107,130
Rb
Abl
HDAC
E2F4,5
DP-1,2
E2F1,2,3
DP-1,2
Cdc6
Cdc45
ORC
MCM
Cdc7
Dbf4
Rb
Wee
Myt1
APC/C
Cdh1
Cdc14
Bub2
MEN
ORC (Origin Recognition Complex)
MCM (Mini-Chromosome Maintenance) complex
Orc1
Orc2
Orc3
Orc4
Orc5
Orc6
Mcm2
Mcm3
Mcm4
Mcm5
Mcm6
Mcm7
DNA
S-phase proteins, CycE
DNA
DNA biosynthesis
G1
S
G2
M
04110 11/15/18
(c) Kanehisa Laboratories

(c)

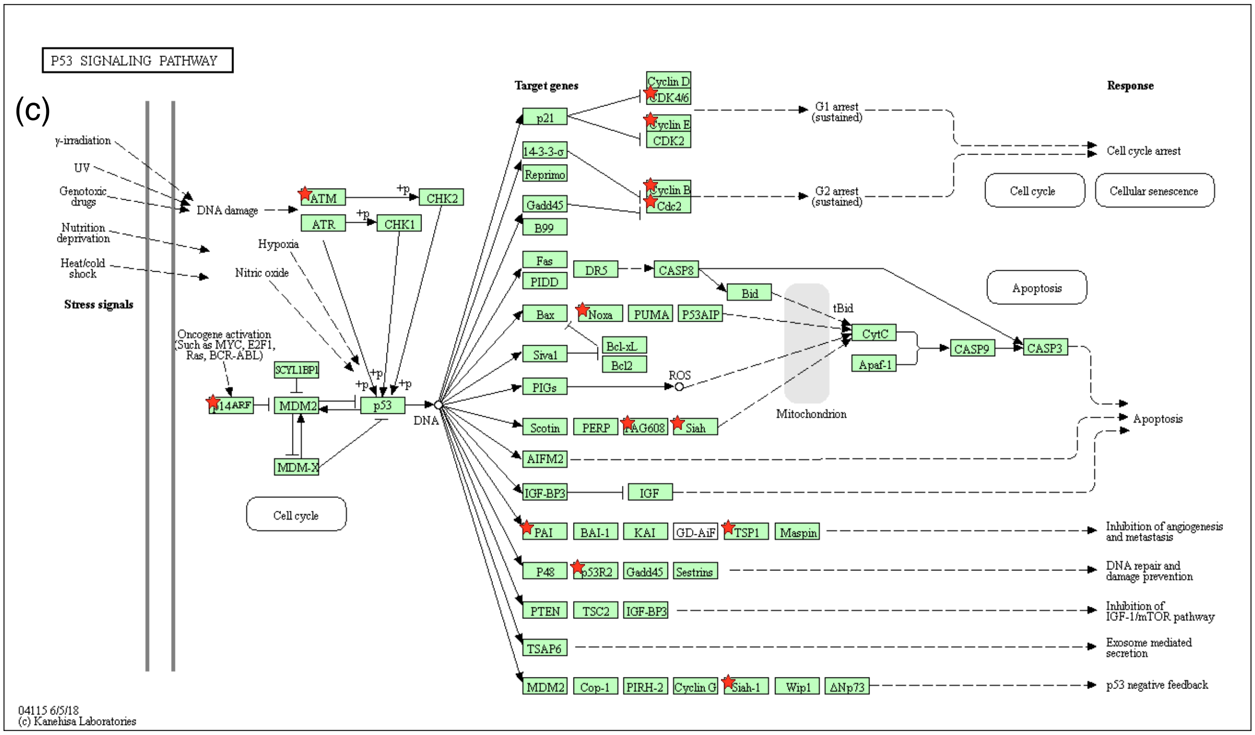
P53 SIGNALING PATHWAY
γ-irradiation
UV
Genotoxic drugs
Nutrition deprivation
Heat/cold shock
Stress signals
DNA damage
Hypoxia
Nitric oxide
Oncogene activation (Such as MYC, E2F1, Ras, BCR-ABL)
ATM
CHK2
ATR
CHK1
SCYL1BP1
p14ARF
MDM2
p53
MDM-X
DNA
Cell cycle
Target genes
p21
14-3-3-σ
Reprimo
Gadd45
B99
Cyclin D
CDK4/6
Cyclin E
CDK2
Cyclin B
Cdc2
G1 arrest (sustained)
G2 arrest (sustained)
Response
Cell cycle arrest
Cell cycle
Cellular senescence
Fas
PIDD
DR5
CASP8
Bid
Apoptosis
Bax
Noxa
PUMA
P53AIP
tBid
CytC
Apaf-1
CASP9
CASP3
Siva1
Bcl-xL
Bcl2
PIGs
ROS
Scotin
PERP
PAG608
Siah
Mitochondrion
Apoptosis
AIFM2
IGF-BP3
IGF
PAI
BAI-1
KAI
GD-AiF
TSP1
Maspin
Inhibition of angiogenesis and metastasis
P48
p53R2
Gadd45
Sestrins
DNA repair and damage prevention
PTEN
TSC2
IGF-BP3
Inhibition of IGF-1/mTOR pathway
TSAP6
Exosome mediated secretion
MDM2
Cop-1
PIRH-2
Cyclin G
Siah-1
Wip1
ΔNp73
p53 negative feedback
04115 6/5/18
(c) Kanehisa Laboratories

(d)

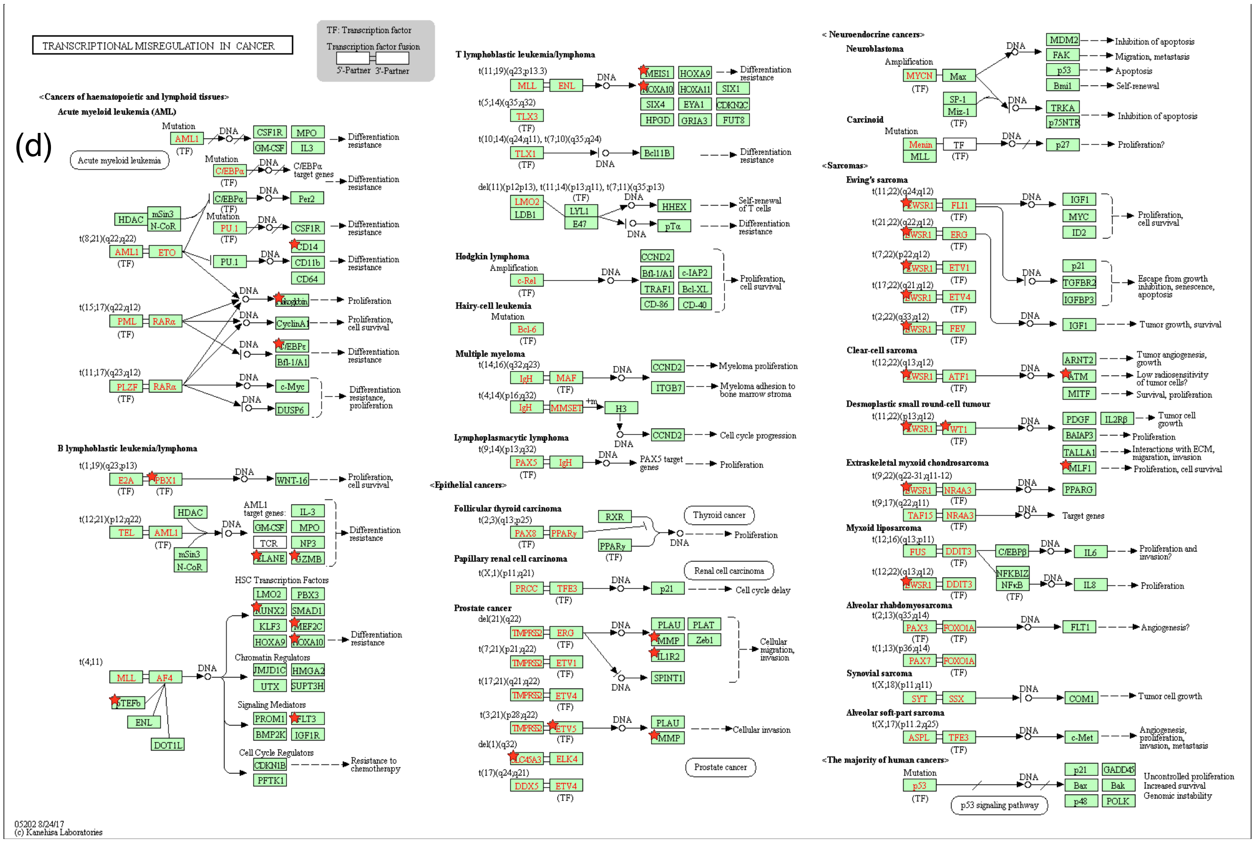

KEGG Pathways. Four KEGG Pathways were identified as enriched, **(a)** Hematopoeitic Cell Lineage, **(b)** Cell Cycle, **(c)** P53 Signaling Pathway and **(d)** Transcriptional Misregulation in Cancer [1-3]. Identified members are marked with a red asterisk.

Supplementary Figure S4: Classification of AML based on 974 DEPS.

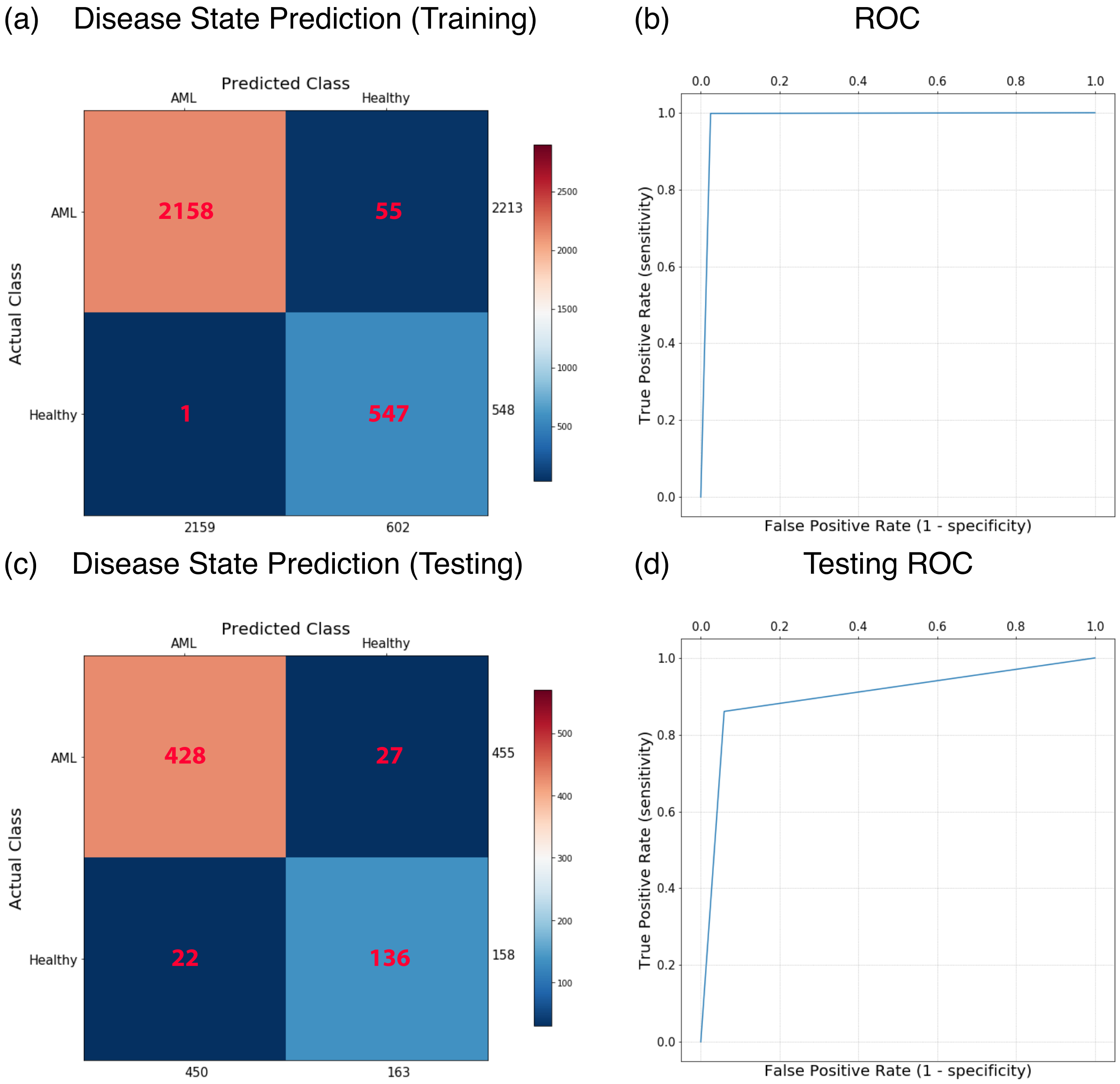

Classification of AML based on 974 DEPS. The 974 DEPS were used to train a KNN model. The results from training (a) and testing (b) show high accuracy and precision. See Supplementary Table S1 for model evaluation statistics.

Supplementary Figure S5: Identification of minimum set of DEPS for KNN model.

(a) Training statistics

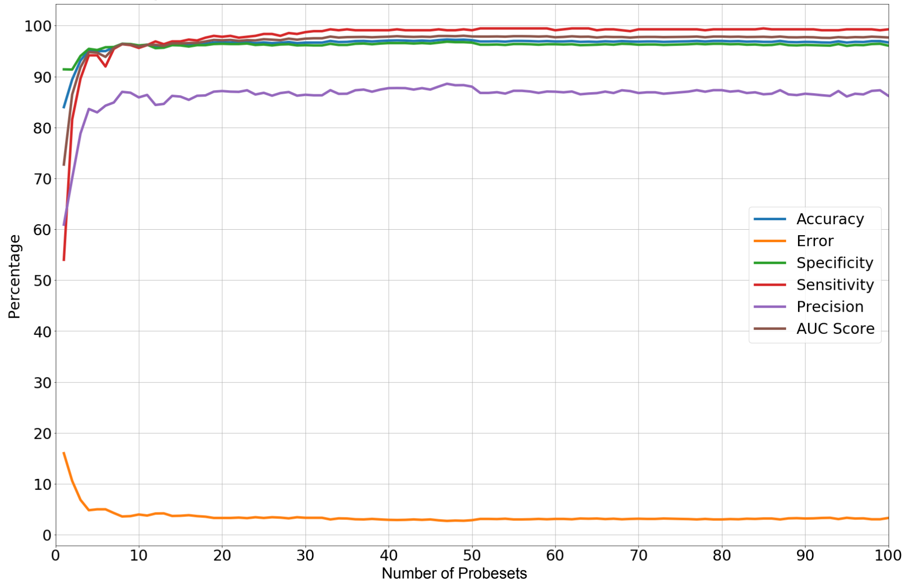

(b) Testing statistics

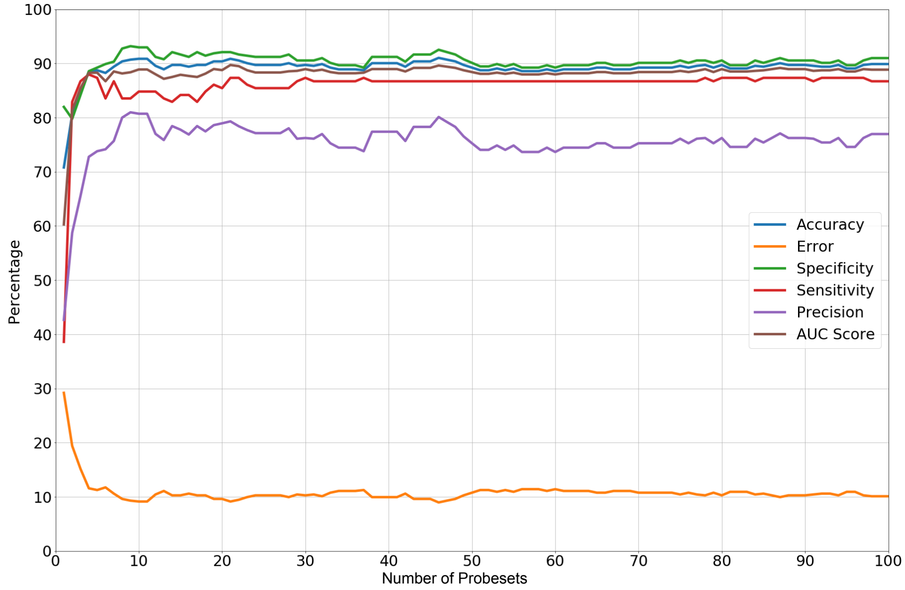

Identification of minimum set of DEPS for KNN model. The *n* top DEPS by expression (ranked by absolute value of effect size) were used to train, with n incremented by one in every iteration. Based on the results from (a) training the model and (b) testing on the covariate datasets, n=10 was identified as a candidate for the minimal DEPS set to use for predicting health status.

Supplementary Figure S6: Classification of AML based on top 10 DEPS base on absolute effect size.

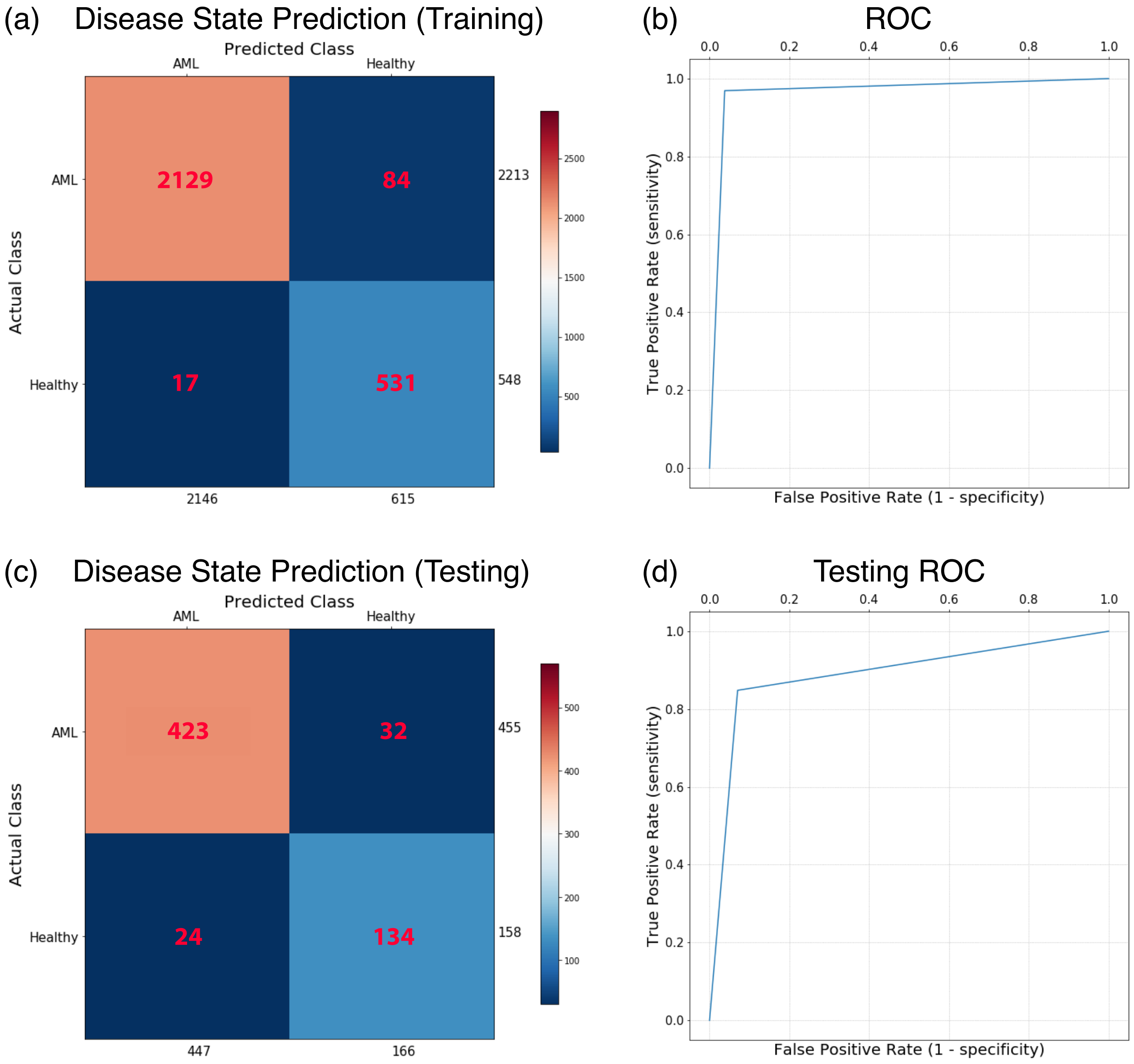

Classification of AML based on top 10 DEPS base on absolute effect size. The top 10 DEPS ranked by effect size (difference in the means) for disease were used to train a KNN model. The results from training (a) and testing (b) show high accuracy and precision. See Supplementary Table S1 for model evaluation statistics.

B. Supplementary Tables

Supplementary Tables are provided as sheets within the Supplementary_Tables.xlsx Excel File. These include:

1. ***Supplementary Table S1***. Model Training and Testing Statistics.
2. ***Supplementary Table S2***. Analysis 1: Gene expression analysis of AML disease state.
3. ***Supplementary Table S3***. Analysis 1: Gene enrichment analysis of AML disease state differentially expressed genes.
4. ***Supplementary Table S4***. Analysis 2a. Sex-relevant differential gene expression analysis in AML.
5. ***Supplementary Table S5***. Analysis 2b. Age-dependent differential gene expression analysis in AML.
6. ***Supplementary Table S6***. Analysis 2b. Age-dependent differential gene expression analysis in AML overlaps with DEPS from Analysis 1.

C. Supplementary Files

The following supplementary files are provided:

1. ***Supplementary_Tables.xlsx***: Supplementary Tables Spreadsheets.
2. ***SupplementaryFile1_SampleSourceClassification.xlsx***: Logistic Regression Training and Testing Results for Sample Source Classification.
3. ***SupplementaryFile2_SexClassification.xlsx***: Logistic Regression Training and Testing Results for Sex Classification.
4. ***SupplementaryFile3_PRISMAFlowChart:*** Data Curation Flow Chart

D. Online Data Availability

Supplementary data, tables, figures and files are available online at https://doi.org/10.5281/zenodo.3257786

	File Name	Description
1	AML_and_Healthy_archive_with_predicted_sex_and_sample_source_for_ANOVA_with_age_groups_and_with_shorter_ID_REF_for_U133AB.csv	Information of 2,761 cases (2,213 AML and 548 healthy) in our analysis: age, sex, sample source, and disease state
2	All_613_AML_and_Healthy_with_44754_probsets_RMA_Normalized_Log2Trans_Zscore_Standardized_Transposed_Data.csv	Control data used for ComBat batch correction
3	All_2761_AML_and_Healthy_with_44754_probsets_RMA_Normalized_Log2Trans_Zscore_Standardized_Transposed_Data.csv	2,761 normalized gene expression data of 44,754 probe sets
4	All_2761_Corrected_for_All_Factors_SampleSource_DiseaseState_Batch_Datasetwise_2213_AML_1st_548_Healthy_2nd_and_removed_613_dummy_with_44754_probsets_RMA_Normalized_Log2Trans_Zscore_Standardized_Transposed_Data_with_shorter_ID_REF_for_U133AB.csv	2,761 batch corrected gene expression
5	ANOVA_P_Values_For_All_2761_Corrected_for_All_Factors_SampleSource_DiseaseState_Batch_Datasetwise_2213_AML_1st_548_Healthy_2nd_and_removed_613_dummy_with_44754_probsets_RMA_Normalized_Log2Trans_Zscore_Standardized_Transposed_Data.csv	ANOVA result for all 2,761subjects
6	Tukey_DiseaseState_P_Values_Corrected_for_All_Factors_SampleSource_DiseaseState_Batch_Datasetwise_multiplied_diff_by_1_corrected.csv	Tukey's Honest Significant Difference test result for AML vs healthy
7	Tukey_Sex_P_Values_Corrected_for_All_Factors_SampleSource_DiseaseState_Batch_Datasetwise.csv	Tukey's Honest Significant Difference test result for female vs male
8	Tukey_SampleSource_P_Values_Corrected_for_All_Factors_SampleSource_DiseaseState_Batch_Datasetwise.csv	Tukey's Honest Significant Difference test result for bone marrow vs normal blood
9	Tukey_Age_P_Values_Corrected_for_All_Factors_SampleSource_DiseaseState_Batch_Datasetwise_with_Probesets_as_Rows_Name.csv	Tukey's Honest Significant Difference test result for age groups
10	1956_Dependent_Data_Subjects_with_Sex_Info_from_all_2761_all_2761_expression_arrays_after_BECorrection	Gene expression dependent data for sex classification (1956 data points)
11	1956_Target_Data_For_Dependent_Data_Subjects_with_Sex_Info_from_all_2761_expression_arrays_after_BECorrection.csv	Gene expression target data for sex classification (1956 data points)
12	805_Testing_Data_Subjects_with_No_Sex_Info_from_all_2761_expression_arrays_after_BECorrection.csv	Gene testing for sex classification (805 data points)
13	2024_Dependent_Data_Subjects_with_SampleSource_Info_from_all_2761_expression_arrays_after_BECorrection.csv	Gene expression dependent data for sample source classification (2024 data points)
14	2024_Target_Data_For_Dependent_Data_Subjects_with_SampleSource_Info_from_all_2761_expression_arrays_after_BECorrection.csv	Gene expression target data for sample source classification (2024 data points)

	File Name	Description
15	737_Testing_Data_Subjects_with_No_SampleSource_Info_from_all_2761_expression_arrays_after_BECorrection	Gene testing for sample source classification (737data points)
16	GPL570_HG-U133_Plus_2.txt	Gene/probe set conversion annotation file for Affymetrix GPL570 array
	SUPPLEMENTARY FILES	
17	***SupplementaryInformation***	Manuscript Supplementary Information
18	***Supplementary_Tables.xlsx***	Supplementary Tables Spreadsheets
19	***SupplementaryFile1_SampleSourceClassification.xlsx***	Logistic Regression Training and Testing Results for Sample Source Classification.
20	***SupplementaryFile2_SexClassification.xlsx***	Logistic Regression Training and Testing Results for Sex Classification.
21	***SupplementaryFile3_PRISMAFlowDiagram.doc***	Data Curation Flow Chart

SUPPLEMENTARY REFERENCES

1 Kanehisa, M., Furumichi, M., Tanabe, M., Sato, Y. & Morishima, K. KEGG: new perspectives on genomes, pathways, diseases and drugs. *Nucleic Acids Res* **45**, D353-D361, doi:10.1093/nar/gkw1092 (2017).

2 Kanehisa, M. & Goto, S. KEGG: kyoto encyclopedia of genes and genomes. *Nucleic Acids Res* **28**, 27-30 (2000).

3 Kanehisa, M., Sato, Y., Kawashima, M., Furumichi, M. & Tanabe, M. KEGG as a reference resource for gene and protein annotation. *Nucleic Acids Research* **44**, D457-D462, doi:10.1093/nar/gkv1070 (2016).
